# History of the taxonomy of Acinetobacter: the emergence of hospital-adapted species of global health concern

**DOI:** 10.1099/ijsem.0.006983

**Published:** 2025-12-05

**Authors:** Nabil Karah, Alexandr Nemec, Bernt Eric Uhlin

**Affiliations:** 1 Department of Molecular Biology and Umeå Centre for Microbial Research (UCMR), Umeå University, 901 87 Umeå, Sweden; 2 Laboratory of Bacterial Genetics, Centre for Epidemiology and Microbiology, National Institute of Public Health, Šrobárova 48, 100 00 Prague 10, Czechia; 3 Department of Medical Microbiology, Second Faculty of Medicine, Charles University and Motol University Hospital, V Úvalu 84, 150 06 Prague 5, Czechia

**Keywords:** antimicrobial resistance, bacterial genomics, eco-evolutionary dynamics, hospital-acquired infections

## Abstract

The genus *Acinetobacter* is widely distributed in nature. A key step in the evolutionary trajectory of *Acinetobacter* is the diversification of species that are well adapted to human-made environments, particularly hospitals, including those capable of causing infection outbreaks. The temporal dynamics, routes and mechanisms of such nosocomial adaptations remain to be elucidated. This review provides a comprehensive description of 162 *Acinetobacter* isolates collected between 1910 and 1970 under various taxonomic names, which may facilitate a more detailed exploration of the pathogenic and nosocomial shifts of *Acinetobacter*. Genomic analysis of these isolates can help reveal the earliest traits of antimicrobial resistance and detect initial genetic events, thereby allowing hypotheses on driving factors that may have preceded the extensive use of modern antimicrobials. Through comparison with contemporary isolates, the evolutionary events and ecological processes that have shaped the phylogeny of today’s highly successful *Acinetobacter* strains can be mapped, and their arsenals of antimicrobial resistance determinants and virulence factors can be tracked chronologically.

**Impact Statement**

*Acinetobacter* has increasingly been recognized as a key opportunistic pathogen causing difficult-to-treat nosocomial infections worldwide. In this review, we reassessed and redescribed the taxonomy of all the *Acinetobacter* isolates that were collected before 1970 and are still available in national culture collections. Insights into these historical *Acinetobacter* isolates facilitate a more precise analysis of the evolution of the genus *Acinetobacter*. This analysis contributes to a better understanding of the nosocomial adaptation of *Acinetobacter baumannii* and supports ongoing efforts to control the emergence of new hospital-adapted *Acinetobacter* species.

## Introduction

The genus *Acinetobacter* is a taxonomically and phylogenetically well-defined member of the family *Moraxellaceae* [1]. It includes bacteria that are Gram-negative, coccobacillary, mesophilic, strictly aerobic, nonfastidious, non-spore-forming, non-flagellated, catalase-positive and oxidase-negative [2,3]. Most strains grow in defined mineral media containing a single carbon and energy source (most commonly acetate), with ammonium or nitrate salts or one of several common amino acids as the nitrogen source [2]. Cells are typically rod-shaped in the exponential phase of growth but often become coccoid in the stationary phase [4]. They may also appear pleomorphic, including filaments without internal division septa [5,6]. Overall, no single metabolic test can reliably distinguish isolates of the genus *Acinetobacter* from other similar nonfermenting Gram-negative bacteria [7]. The *Acinetobacter* genome consists of a single circular chromosome, ranging in size between 2.6 and 4.7 Mb, and a strain-dependent set of extrachromosomal elements. The ratio of guanine and cytosine to the total amount of nucleotides in the genomes of *Acinetobacter* varies between 34.9 and 46.9%, with a median of 39.6 or 40.2% [3,8].

*Acinetobacter* is a ubiquitous genus that is highly diverse in its taxonomic structure and metabolic characteristics. Both culture-based and metagenomic studies have shown that *Acinetobacter* isolates are particularly abundant in soil, host-associated environments, pristine aquatic environments and organic-rich aquatic environments [3,9]. Their presence in a variety of food products and in atypical locations has also been noted [10,11]. Phylogenomic analysis of the core genome revealed that the genus *Acinetobacter* has undergone extensive evolutionary diversification, and it is estimated to be as old as the entire family *Enterobacteriaceae*, i.e. about 500 megaannum [8]. The evolutionary age, phylogenetic diversification and ecological ubiquity are reflected in the abundance of *Acinetobacter* species. As of September 2025, the genus included 87 species with validly published correct names [12], with many additional taxonomically unique provisional taxa and strains awaiting taxonomic clarification [3,13]. Importantly, the misidentification and misclassification of *Acinetobacter* isolates to the species level is a common issue [14,15]. For example, ~3% (6 out of 214) of the *Acinetobacter* genomic data deposited at the U.S. National Center for Biotechnology Information (https://www.ncbi.nlm.nih.gov/) were found to be misclassified under incorrect species names [15]. Although the genus name *Acinetobacter* was coined as early as 1954 [16], organisms now classified as *Acinetobacter* were known under different names since the beginning of the 20^th^ century. A fundamental change in bacterial nomenclature was taken on 1 January 1980, when the Approved List of Bacterial Names came into effect [17]. This list contained valid scientific names with formal standing in the nomenclature, carefully selected to eliminate the nomenclatural chaos that had arisen in previous decades and to emphasize the importance of an approach based on newly defined nomenclatural rules [18,19]. All other names, i.e. those that appeared in the literature before January 1980 but were not included in the Approved List, became invalid. Nevertheless, it is of great value to decipher the relationships of current species names to their invalid predecessors and obsolete synonyms. This is particularly important for several *Acinetobacter* species that have suffered from a long history of extensive taxonomic change [20]. The aim of this article is to compile data on *Acinetobacter* isolates collected before 1970 and to trace the changes in their taxonomy and nomenclature to the currently used names. All the species names used in our manuscript, including their abbreviated forms, are summarized in Table S1 (available in the online Supplementary Material).

## The earliest isolates of *Acinetobacter calcoaceticus*

**Fig. 1.**
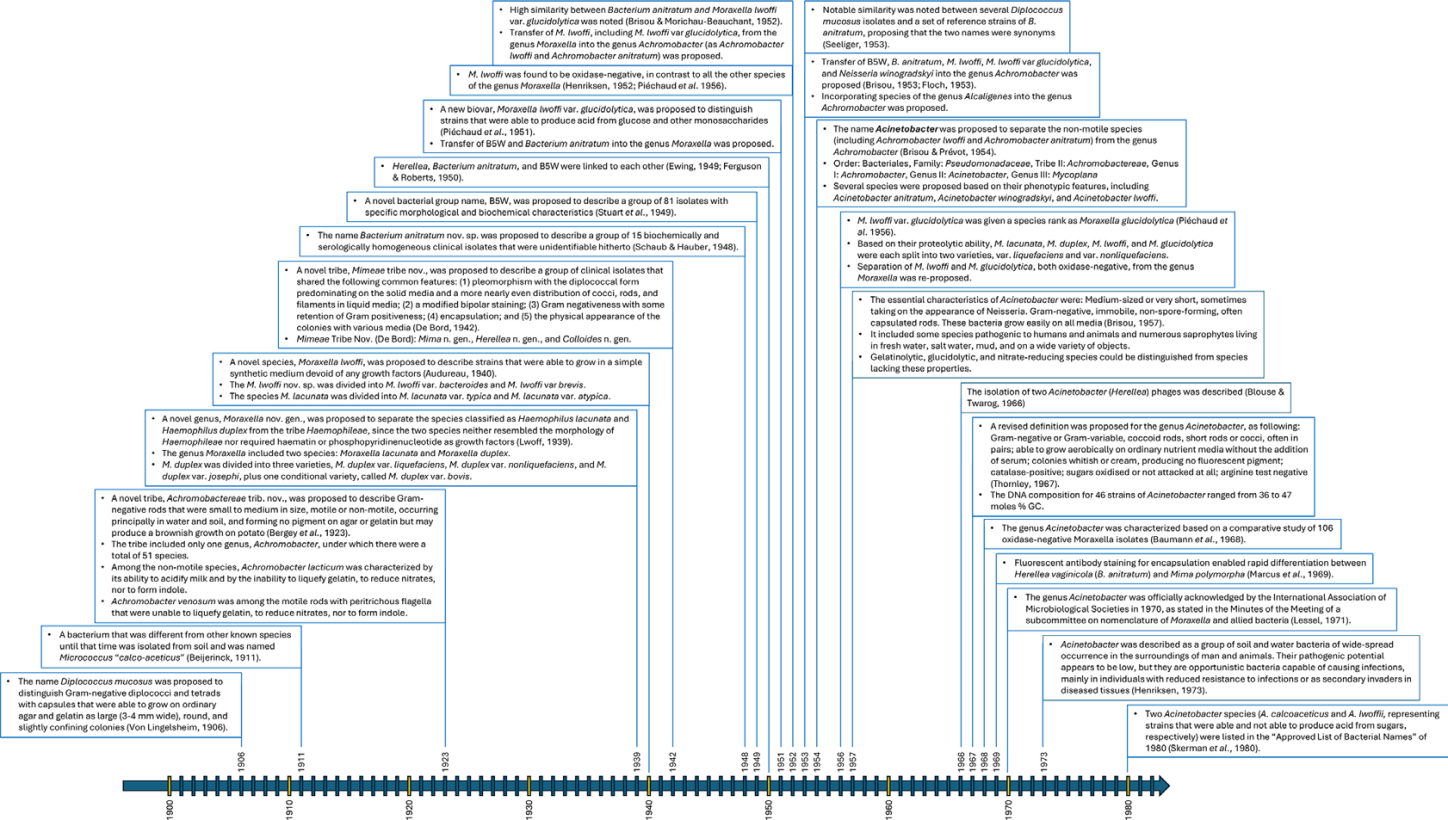
A chronology of the major changes that took place in the history of *Acinetobacter* in the period from 1900 to 1980. Von Lingelsheim, 1906 (reference no. [136]); Beijerinck, 1911 [21]; Bergey *et al*., 1923 [61]; Lwoff, 1939 [34]; Audureau, 1940 [36]; De Bord, 1942 [82]; Schaub & Hauber, 1948 [92]; Stuart *et al*., 1949 [97]; Ewing, 1949 [104]; Ferguson & Roberts, 1950 [100]; Piéchaud *et al*., 1951 [105]; Henriksen, 1952 [33]; Piéchaud *et al*., 1956 [108]; Brisou & Morichau-Beauchant, 1952 [107]; Seeliger, 1953 [137]; Brisou, 1953 [115]; Floch, 1953 [117]; Brisou & Prévot, 1954 [16]; Brisou, 1957 [125]; Blouse & Twarog, 1966 [168]; Thornley, 1967 [93]; Baumann *et al*., 1968 [2]; Marcus *et al*., 1969 [165]; Lessel, 1971 [134]; Henriksen, 1973 [5]; Skerman *et al*., 1980 [17].

In 1911, Dutch microbiologist M. W. Beijerinck reported the isolation of bacteria from soil that were different from all species known at that time (Fig. 1) [21]. The discovered bacterium was named ‘*Micrococcus calco-aceticus*’ since calcium acetate was suitable for its accumulation. One of the ‘*M. calco-aceticus*’ isolates reported by Beijerinck, hereafter called Delft 1, has been preserved until today under various culture collection names (Table 1, 1). In 1968, Delft 1 was nominated as the type strain of *A. calcoaceticus*, which was selected to be the type species of the genus *Acinetobacter* [2]. Delft 1 has since then become a commonly used strain in the taxonomic studies of *Acinetobacter* [17,22, 23]. Analysis of the whole genome of four subcultures of Delft 1 confirmed its classification (GenBank accession numbers: APQI00000000.1, AIEC00000000.1, BBTM00000000.1 and UFSJ00000000.1, corresponding to CIP 81.8^T^, DSM 30006^T^, KCTC 2357^T^ and NCTC 12983^T^, respectively). The colonies of NCIMB 10694^T^, another subculture of Delft 1, were described as opaque-translucent [24]. Similarly, the subculture of LMG 1046^T^ contained two stable colony types [25], which was also reported for ATCC 23055^T^ [26]. Cells of the two ATCC 23055^T^ colony types showed a number of micromorphological and phenotypic differences, while their thermal denaturation curves and electrophoretic protein patterns were almost identical [26].

**Table 1.** Basic isolation and deposition data of the strains, including all their names and designations

No.	Strain*	Year of isolation	Country of isolation	Source of sample	Owner or prime depositor
*Acinetobacter calcoaceticus* **=Genospecies 1**					
1	ATCC 23055^T^, Baumann 46 (Doudoroff 46), BCRC 17328^T^, Bouvet and Grimont 1, CAIM 17^T^, CCT 0050^T^, CCUG 12804^T^, CECT 441^T^, CIP 81.8^T^ (CIP 81.08^T^), CNCTC 7610^T^, **Delft 1** (Beijerinck 1, LMD 1), Delft 317 (LMD 317), Dijkshoorn 75, Dijkshoorn serial no. 1, DSM 30006^T^, E.111.9.4.1., FIRDI 351, IAM 12087^T^, JCM 6842^T^, Johnson and Sneath J25, Juni 46, KCTC 2357^T^, LMG 1046^T^, LMG 10511, NBIMCC 3730^T^, NBRC 13718 (IFO 13718), NCCB 22016^T^ (LMD 22.16), NCCB 89014 (LMD 89.14), NCCB 95114 (LMD 95.114), NCCB 95116 (LMD 95.116), NCIMB 10694^T^ (NCIB 10694^T^), NCTC 12983^T^, NIPH 2245^T^, NRRL B-65370^T^, NZRM 3185^T^, RUH 2201^T^, VTT E-981117^T^	≤1911	Unknown	Soil	M.W. Beijerinck
2	ATCC 19638, ATCC 23322, **Delft L360** (LMD L360), Hugh 2414 (RH2414), NCIMB 9029 (NCIB 9029), Sahl TG19588	≤1911	Unknown	Soil	M.W. Beijerinck
3	ATCC 19639, **Delft L361** (LMD L361), Dijkshoorn 74, Dijkshoorn serial no. 2, E.III.9.4.2., Hugh 2415, NCCB 22017 (LMD 22.17), RUH 54	≤1911	Unknown	Soil	M.W. Beijerinck
4	ATCC 17902, Baumann 90, BCRC 15992, Bouvet and Grimont 2, CCM 4503, CDC KC774 (NCDC KC774), **CIP 66.32**, CNCTC 5168, Hugh 2269 (RH 2269), LMG 992, Sahl TG19585	≤1952	France	Soil	Girard and M. Lemoigne
5	ATCC 23236, Baumann 61, BCRC 11562, Bouvet and Grimont 3, CCUG 3352, CIP 66.33, CIP 68.32, DSM 1139, Hugh 2338 (RH 2338), **Kallio H.O.1** (Rey HO-1), LMG 1056, NBRC 12552 (IFO 12552), NCAIM B.02622, NCCB 82032 (LMD 82.32), NCIM 2890, NCIMB 9205 (NCIB 9205), Sahl TG19593	≤1959	Unknown	Soil	R.E. Kallio
*Acinetobacter baumannii* **=Genospecies 2**					
6	**Allen 4H**, ATCC 23220, CIP 68.38, Dijkshoorn AC51, DSM 30011, Johnson and Sneath J37, LMAU A4, LMG 1049, NCCB 82027 (LMD 82.27), NCDO 709, NCIM 2433, NCIMB 8208 (NCIB 8208), NRRL B-551, Thornley 3	≤1944	Unknown	Guayule shrubs	M.J. Thornley
7	**Allen 6H**, CCM 70, LMG 1052, NCDO 710, NCIMB 8209 (NCIB 8209), NRRL B-552	≤1944	Unknown	Guayule shrubs	Naghski
8	ATCC 9955, Baumann 49, Bouvet and Grimont 21, CDC KC738 (NCDC KC738), CIP 70.24, CNCTC 6170, CNCTC Mi 5/65, **Deacon 6-561**, Hugh 483 (RH 483), LMG 984, Thornley 4	≤1944	Italy	Cerebrospinal fluid	W.E. Deacon
9	Baumann 52, **NCTC 7364**, strain Carr	≤1947	UK	Unknown	A.P. Peeney
10	**NCTC 7412**, strain Jones	≤1948	UK	Urine	A.P. Peeney
11	**NCTC 7422**, strain Ayers	≤1948	UK	Burn, leg	H.W. Dalton
12	ATCC 19606^T^, BCRC 10591^T^ (CCRC 10591^T^), Bouvet and Grimont 4, CCT 1432^T^, CCUG 19096^T^, CCUG 57815^T^, CIP 70.34^T^, CNCTC 6168^T^, CNCTC B 5/78^T^, Dijkshoorn serial no. 35, Dijkshoorn AC1099^T^, DSM 30007^T^, DSM 6974^T^, FIRDI 591, Hugh 2208 (RH 2208), IAM 12088^T^, JCM 6841^T^, KCTC 2508^T^, LMG 1041^T^, LMG 10545^T^, NBRC 109757^T^, NCAIM B.01930^T^, NCAIM B.02074^T^, NCCB 85021^T^ (LMD 85.21^T^), NCCB 96002^T^ (LMD 96.2^T^), NCCB 96128^T^ (LMD 96.128^T^), NCIM 5663, NCIMB 12457^T^ (NCIB 12457^T^), NCTC 12156^T^, NIPH 501^T^, NRRL B-65371^T^, NZRM 3289^T^, RUH 3023^T^, **Schaub 81**, VTT E-981116^T^	≤1948	USA	Urine	I.G. Schaub
13	ATCC 19187, Canad-226 93, Johnson and Sneath J38, Hugh 2317 (RH 2317), LMG 1037, NCIMB 9212 (NCIB 9212), Ontario B11, **Schaub 93**, Sneath D.411, Thornley 36	≤1948	USA	Urine	I.G. Schaub
14	ATCC 15308, BCRC 15318 (CCRC 15318), CCEB 765, CCM 5593, CCUG 33549, CECT 452, CIP 64.8, Dijkshoorn 78, Dijkshoorn AC1063, Dijkshoorn AC1213, Dijkshoorn serial no. 15, DSM 30008, Ferguson B5W 101, Hugh 2197 (RH 2197), IMI 347320, IMI B03456, Lampe RF34, LMG 991, LMG 10525, NBIMCC 553, NCCB 85020 (LMD 85.20), NCCB 85025 (LMD 85.25), NCCB 95117 (LMD 95.117), NCTC 7844, RUH 1063, **Schaub Biol. 1** (Schaub and Hauber Biol. 1), USCC 1612	≤1948	USA	Urine	I.G. Schaub
15	ATCC 19568, Bouvet and Grimont 15, CIP 70.35, Hugh 2315 (RH 2315), LMG 1057, NCIMB 9214 (NCIB 9214), Ontario B9, **Schaub Biol. 2**, Sneath D.409, Thornley 34	Around 1948	USA	Urine	I.G. Schaub
16	ATCC 17945, Bouvet and Grimont 9, CDC KC732, CIP 70.22, Hugh 2385 (RH 2385), Lautrop A1, **Stuart B5W 3777**	Around 1949	Unknown	Unknown	C.A. Stuart
17	ATCC 15151, BCRC 15884 (CCRC 15884), Billing BT9, Bouvet and Grimont 11, CDC KC731 (NCDC KC731), CIP 70.10, **Ferguson B5W 72**, LMG 990, NCIMB 9300 (NCIB 9300), Ontario B24, Stuart H606, Thornley 38	≤1950	Unknown	Clinical sample	C.A. Stuart
18	ATCC 15149, Billing BT8, Bouvet and Grimont 6, CDC KC729 (NCDC KC729), CIP 70.8, CNCTC 6166, CNCTC B 3/65, **Ferguson B5W 71**, Hugh 2226 (RH 2226), NBIMCC 554, NCIMB 9299 (NCIB 9299), Stuart H1166	≤1950	Unknown	Clinical sample	I.G. Schaub
19	ATCC 15150, Billing BT2, Bouvet and Grimont 23, CDC KC730 (NCDC KC730), CIP 70.9, **Ferguson B5W 3**, LMG 989, NCCB 82047 (LMD 82.47), NCIMB 9293 (NCIB 9293), Ontario B16, Thornley 37	≤1950	Unknown	Urine	W.W. Ferguson
20	ATCC 17978, Bouvet and Grimont 8, CDC KC755 (NCDC KC755), CIP 53.77, DSM 105126, LMG 1025, **Piéchaud strain Rochefort**, Piéchaud 5377	≤1951	Unknown	Cerebrospinal fluid	Piéchaud
21	CIP 53.79, **Piéchaud strain Gaillard**	≤1951	Unknown	Whitlow	Piéchaud
22	**CIP 53.66**	≤1953	Unknown	Cerebrospinal fluid	Piéchaud
23	ATCC 17957, Bouvet and Grimont 14, CDC KC734 (NCDC KC734), CIP 70.21, Hugh 2386 (RH 2386), LMG 1014, Lautrop A194, **Seeliger D5/62**	≤1953	Germany	Unknown	Seeliger
24	CIP 54.147, **Piéchaud strain Valentin**	≤1954	Unknown	Cerebrospinal fluid	Piéchaud
25	Baumann 70, CIP 54.97, **Piéchaud strain de Croy**	≤1954	Unknown	Pulmonary abscess	Y.R. Chabbert
26	ATCC 19003, BCRC 15886 (CCRC 15886), Bouvet and Grimont 18, CDC KC740 (NCDC KC740), CIP 70.28, **Courtieu 57.071.066**, Hugh 2390 (RH 2390), Juni 42, LMG 1034	1957	Unknown	Cerebrospinal fluid	A.L. Courtieu
27	**499**, 499 (M4), ATCC 14293, Hugh 2421 (RH 2421)	≤1958	USA	Clinical sample	S.G. Cary
28	CCUG 2488, Enjalbert 88.504, **Kambou 88.504**, LMG 1067, NCCB 82037 (LMD 82.37), NCIMB 9306 (NCIB 9306)	≤1959	Unknown	Unknown	Enjalbert
29	**Lautrop B5W**, LMG 1164, NCTC 10292, USCC 1615	≤1961	USA	Unknown	A. Stuart
30	ATCC 17904, Baumann 65, Bouvet and Grimont 19, CCM 2355, CDC KC722 (NCDC KC722), CIP 64.1, CCUG 890, CNCTC 7769, Dijkshoorn serial no. 25, HIH 3516/60, Hugh 2211 (RH 2211), Johnson and Sneath J35, Lautrop AB1181, LMG 994, LMG 10535, NCTC 10303, RUH 2209, **Stenzel and Mannheim 3516/60**, Torry 7-36	≤1962	Germany	Urine	Stenzel
31	ATCC 17961, Baumann 55, Bouvet and Grimont 5, Juni 29, CDC 7788, CDC KC741 (NCDC KC741), CIP 70.32, Hugh 2424 (RH 2424), Juni 29, **Kass 13860,** LMG 1016, USCC 1435	≤1964	Unknown	Blood	Kass
32	ATCC 17959, Baumann 100, Bouvet and Grimont 13, **CDC 7827**, CDC KC742 (NCDC KC742), CIP 70.33, Juni 28	≤1964	Unknown	Unknown	J.E. Johnson
*Acinetobacter pittii* **=Genospecies 3**					
33	**CIP 52.90**	1952	France	Throat	Second
34	ATCC 19004^T^, Bouvet and Grimont 25, CCUG 61664^T^, CDC KC739 (NCDC KC739), CIP 70.29^T^, **Courtieu 57.071.228**, Dijkshoorn serial no. 55, DSM 21653^T^, DSM 25618^T^, Hugh 2425 (RH 2425), Juni 43, KCTC 52595^T^, LMG 1035^T^, NIPH 519^T^, RUH 2206^T^	1957	Unknown	Cerebrospinal fluid	A.L. Courtieu
35	ATCC 17922, Bouvet and Grimont 26, **Brisou 12**, CCUG 2490, CDC KC726 (NCDC KC726), CIP 70.15, Hugh 2382 (RH 2382), NCCB 82029 (LMD 82.29), NCIMB 9017 (NCIB 9017)	≤1957	Unknown	Unknown	Brisou
*Acinetobacter haemolyticus* **=Genospecies 4**					
36	CIP 53.112, **Piéchaud strain n° 1 (Lagarrigue**)	≤1953	Unknown	Sputum	Piéchaud
37	ATCC 17977, Bouvet and Grimont 34, CDC KC768, CIP 53.116, LMG 1024, **Piéchaud strain n° 2 (Zlotagora I**)	≤1953	Unknown	Rectal ulceration	Piéchaud
38	CIP 53.67, **Piéchaud strain n° 3 (Zlotagora II**)	≤1953	Unknown	Rectal ulceration	Piéchaud
39	CIP 53.111, **Piéchaud strain n° 4 (Berthon**)	≤1953	Unknown	Faeces	Piéchaud
40	CIP 53.71, **Piéchaud strain n° 7 (Orcival**)	≤1953	Unknown	Postoperative meningitis	Piéchaud
41	CIP 53.115, **Piéchaud strain n° 8 (Dujarric**)	≤1953	Unknown	Urine	Piéchaud
42	**CIP 53.143**	≤1953	Unknown	Conjunctivitis	Piéchaud
43	CIP 55.105, **Piéchaud strain n° 10**	1955	Unknown	Leg ulceration	Piéchaud
44	ATCC 17913, Bouvet and Grimont 37, **Billing A3;ST3**, CDC KC715 (NCDC KC715), CIP 70.14, Hugh 2378 (RH 2378), LMG 1001, NCIMB 9116 (NCIB 9116)	≤1955	Unknown	Unknown	Billing
45	ATCC 19194, Bouvet and Grimont 39, CDC KC737, CIP 70.27, **Courtieu 55.071.323**, Hugh 2389 (RH 2389), Juni 44, LMG 1241	1955	Unknown	Nose	A.L. Courtieu
46	ATCC 19002, Bouvet and Grimont 40, CDC KC735 (NCDC KC735), CIP 70.26, **Courtieu 57.073.192**, Juni 41, LMG 1033, Sahl TG19602	1957	Unknown	Ocular pus	A.L. Courtieu
47	ATCC 17906^T^, Baumann 40, Bouvet and Grimont 32, BCRC 14852^T^, CCM 2358^T^, CCUG 888^T^, CDC KC719 (NCDC KC719), CIP 64.3^T^, Dijkshoorn AC1217, Dijkshoorn serial no. 60, DSM 6962^T^, HIH 2446/60, Hugh 2213 (RH 2213), Lautrop AB1183, **Stenzel and Mannheim 2446/60**, Johnson and Sneath J32, Juni 16, KCTC 12404^T^, LMG 996^T^, LMG 1236, LMG 10570, MTCC 9819^T^, NBRC 109758^T^, NCCB 85026^T^ (LMD 85.26), NCIMB 12458^T^, NCTC 10305^T^, NCTC 12155, NZRM 3971^T^, RUH 2215^T^, Sahl TG19599, Torry 7–38, USCC 1605, VTT E-981119^T^	≤1962	Germany	Sputum	Stenzel
48	ATCC 17907, Baumann 44, Bouvet and Grimont 38, CCM 2359, CCUG 891, CDC KC718 (NCDC KC718), CIP 64.4, HIH 2181/60, Johnson and Sneath J31, Lautrop AB1184, LMG 997, LMG 1237, NBIMCC 148, NCTC 10306, **Stenzel and Mannheim 2181/60**, Torry 7-39, USCC 1604	≤1962	Germany	Pus	Stenzel
49	**9458**, ATCC 27244, Sahl TG21157	≤1963	USA	Cervix	W.B. Cherry
*Acinetobacter junii* **=Genospecies 5**					
50	CIP 55.115, **Rubinsten strain 1**	1947	Unknown	Faeces	Rubinsten
51	**CIP 51.14**	1951	France	Guinea pig, spleen	Gaillard
52	**CIP 51.35**, strain Ouazeme	≤1951	France	Blood	Unknown
53	ATCC 19001, CDC KC736 (NCDC KC736), CIP 70.25, **Courtieu 56.073.110**, LMG 1032	1956	Unknown	Bronchial secretions	A.L. Courtieu
54	ATCC 17908^T^, Baumann 10, Bouvet and Grimont 42, CDC KC716 (NCDC KC716), CL 333/86, DSM 6964^T^, CCM 2376^T^, CCUG 889^T^, CIP 64.5^T^, Dijkshoorn serial no. 63, HIH 2723/59, Johnson and Sneath J33, Lautrop AB1185, LMG 998, LMG 10573, KCTC 12416^T^, KCTC 12406^T^, NBIMCC 146^T^, NBRC 109759^T^, NCIMB 12459, NCTC 10307^T^, NCTC 12153, RUH 2228^T^, Sahl TG19608, **Stenzel and Mannheim 2723/59**, Torry 7-40, VTT E-981121^T^, USCC 1602	≤1962	Germany	Urine	Stenzel
*Acinetobacter* **genospecies 6**					
55	ATCC 17979, BCRC 15421, Bouvet and Grimont 46, CCUG 26492, CDC KC754 (NCDC KC754), CIP A165, Dijkshoorn serial no. 68, Hugh 2392 (RH 2392), LMG 1026, LMG 10578, **Piéchaud A165**, Piéchaud strain n° 5, RUH 2867	≤1952	France	Throat	Piéchaud/L. Second
*Acinetobacter johnsonii* **=Genospecies 7**					
56	**ATCC 9036**, Baumann 28, Bouvet and Grimont 58, CIP 68.41, IAM 1517, JCM 20194, Johnson and Sneath J9, LMG 983, NBRC 102197, NCCB 80001 (LMD 80.1), NCDO 715, NCIMB 8154 (NCIB 8154), Thornley 27	≤1936	Unknown	Unknown	B.W. Hammer
57	ATCC 17969, **Audureau strain bacteroides**, Bouvet and Grimont 56, CDC KC763, CIP A171, Juni 31, LMG 1018, Piéchaud A171	≤1940	France	Chronic conjunctivitis	Audureau
58	ATCC 17946, BCRC 15888 (CCRC 15888), Bouvet and Grimont 59, CDC 911, CDC KC733 (NCDC KC733), CIP 70.23, **Lautrop A3**, LMG 1005	≤1953	Unknown	Unknown	I.J. Schuldberg
59	ATCC 17923, Bouvet and Grimont 55, **Brisou 63**, CDC KC723 (NCDC KC723), CIP 70.16, LMG 1002, NCCB 82030 (LMD 82.30), NCIMB 9018 (NCIB 9018)	≤1957	Unknown	Unknown	Brisou
60	CCUG 2486, **Kambou 87.019**, LMG 1064, NCIMB 9302 (NCIB 9302)	≤1959	Unknown	Unknown	Enjalbert
61	ATCC 17909^T^, Baumann 8, BCRC 14853^T^ (CCRC 14853), Bouvet and Grimont 48, CDC KC720 (NCDC KC720), CCUG 19095^T^, CIP 64.6^T^, CL 335/86, Dijkshoorn serial no. 69, DSM 6963^T^, HAMBI 1969^T^, HAMBI 1971^T^, KCTC 12405^T^, Lautrop AB1186, LMG 999^T^, LMG 1238, LMG 10579, NCIMB 12460^T^, NCTC 10308^T^, NCTC 12154, RUH 2231^T^, Sahl TG19605, **Stenzel and Mannheim 3865/60**, Torry 7-41, USCC 1606, VTT E-981120^T^	≤1962	Germany	Duodenum	Stenzel
62	Baumann 19, Bouvet and Grimont 57, CIP 77.14, Johnson and Sneath J28, **Piéchaud strain Tanger H315**	≤1968	France	Blood	Piéchaud
*Acinetobacter pseudolwoffii* **=Genospecies 8**					
63	ANC 4579, Bouvet and Grimont 60, CCT 0052, **CIP 64.10**, CNCTC 7645	Unknown	Unknown	Unknown	Supposed to be from NCTC 5866^T^
64	ANC 4683, ATCC 17925, **Brisou 65**, Bouvet and Grimont 61, CDC KC725 (NCDC KC725), CIP 70.17, LMG 1004, NCIMB 9020 (NCIB 9020), USCC 1472	≤1957	Unknown	Unknown	Brisou
*Acinetobacter lwoffii* **=Genospecies 9**					
65	ATCC 15309^T^, ATCC 17986, BCRC 14855^T^ (needs verification), CCM 5581^T^, CCUG 12805, CCUG 33984^T^, CDC KC765 (NCDC KC765), CECT 453^T^, CIP 110687^T^, CNCTC 6167^T^, CNCTC 4/75, Dijkshoorn 71, Dijkshoorn serial no. 77, DSM 2403^T^, Hugh 2393 (RH 2393), JCM 6840^T^, Johnson and Sneath J27, KCTC 12407^T^, LMG 1029^T^, LMG 1240, LMG 10587, **Lwoff 1**, NBRC 109760^T^, NCAIM B.01101^T^, NCCB 73001^T^ (LMD 73.1), NCCB 83025 (LMD 83.25), NCIMB 12456^T^ (needs verification), NCIMB 13080, NCTC 5866^T^, NIPH 512^T^, NZRM 2581^T^, Pintér and Bende 29, RUH 2219^T^, Thornley 63, VTT E-97855^T^	≤1939	France	Unknown	Lwoff
66	ATCC 17987, CCM 5572, CDC KC767 (NCDC KC767), CIP 70.36, LMG 1030, **Lwoff 2**, NBIMCC 1125, NCTC 5867, Thornley 64, USCC 1453	1939	France	Unknown	Lwoff
67	ATCC 17968, **Audureau strain brevis**, Bouvet and Grimont 65, CDC KC766 (NCDC KC766), CIP A162, LUH 1713, NIPH 2175, Piéchaud A162	≤1940	France	Conjunctivitis	Audureau
68	ATCC 9957, Baumann 25, Bouvet and Grimont 62, CCUG 61662, CDC KC743 (NCDC KC743), CIP 70.31, CNCTC 6172, CNCTC Mi 3/65, **Deacon H-B**, Dijkshoorn 72, Dijkshoorn AC1053, Hugh 482 (RH 482), LMG 985, LUH 1710, NCCB 83024 (LMD 83.24), NCCB 86008 (LMD 86.8), NIPH 2172	≤1945	Italy	Gas gangrene	W.E. Deacon
69	CIP 55.112, **Rubinsten strain 18**	1947	Unknown	Spinal fluid	Rubinsten
70	CIP 55.113, **Rubinsten strain 19**	1947	Unknown	Blood	Rubinsten
71	**NCTC 7976**, Thornley 65	≤1949	Unknown	Unknown	Wheatley
72	ANC 4568, **CIP 51.11**, CNCTC 7857	1951	France	Pleural pus	No information
73	CIP 54.98, **Haguenau 11.976**	≤1954	France	Blood	Haguenau lab
74	CIP 55.102, **Saint Quentin strain 2**	1955	France	Spinal fluid	Chatelain
75	**Brisou 78**, CCUG 2489, LMG 1055, NCIMB 9022 (NCIB 9022), Thornley 5	≤1957	Unknown	Unknown	Brisou
76	ATCC 17910, Bouvet and Grimont 68, CDC KC721 (NCDC KC721), CIP 64.7, Hugh 2217 (RH 2217), Lautrop AB1187, LMG 1000, LMG 1239, NCTC 10309, Sahl TG19636, **Stenzel and Mannheim P790/60**, Torry 7-42, USCC 1607	≤1962	Germany	Urine	Stenzel
77	ATCC 23221, CIP 68.34, DSM 30012, **Klinge 950/56**, LMG 1050, NZRM 2727, Thornley 66	≤1967	Austria	Unknown	Flamm
78	ATCC 17985, CDC KC764 (**NCDC KC764**), CIP 70.20, LMG 1028, NBIMCC 1124	≤1968	Unknown	Unknown	Institut Pasteur
79	ATCC 17984, Bouvet and Grimont 66, CDC KC762 (NCDC KC762), CIP 70.19, **Henriksen strain Lausanne 397**, Juni 36, NIPH 2176, LUH 1714	≤1970	Unknown	Unknown	S.D. Henriksen
*Acinetobacter bereziniae* **=Genospecies 10**					
80	ATCC 17924^T^, BCRC 15423^T^, **Brisou 64**, Bouvet and Grimont 69, CDC KC724 (NCDC KC724), CECT 442^T^, CIP 70.12^T^, CNCTC 7606^T^, Dijkshoorn AC 2224, Dijkshoorn serial no. 92, DSM 25435^T^, Hugh 2380 (RH 2380), KCTC 42001, LMG 1003^T^, LMG 10602, NCCB 82031^T^ (LMD 82.31), NCIMB 9019 (NCIB 9019), NIPH 521^T^, RUH 2224^T^, Thornley 13	≤1957	Unknown	Wound	Brisou
*Acinetobacter guillouiae* **=Genospecies 11**					
81	ATCC 11171^T^, Baumann 94, BCRC 15424 (CCRC 15424), Bouvet and Grimont 73, CCT 1870, CCUG 2491^T^, CIP 63.46^T^, Dijkshoorn serial no. 94, DSM 590^T^, KCTC 23200^T^, LMG 988^T^, NCCB 80002^T^ (LMD 80.2), NCIM 2886, NCIMB 8250 (**NCIB 8250**), RUH 2861, VTT E-981118^T^	≤1951	Unknown	Sewage	W.C. Evans
*Acinetobacter nosocomialis* **=Genospecies 13TU**					
82	ATCC 13695, ATCC 17903, Bouvet and Grimont 29, CCM 5594, CCUG 26488, CDC KC728, CIP 70.11, Dijkshoorn serial no. 106, DSM 30010, Hugh 2210 (HR 2210), LMG 993, NCAIM B.01100, NCTC 8102, RUH 2210, Sahl TG21145, **Stuart A267**, Thornley 1	≤1949	USA	Unknown	C.A. Stuart
*Acinetobacter colistiniresistens* **=Genospecies 13BJ/14TU**					
83	ATCC 17905, Baumann 30, Bouvet and Grimont 82, CCM 2357, CCUG 887, CDC KC717 (NCDC KC717), CIP 64.2, CNCTC 7582, Dijkshoorn serial no. 120, Hugh 2212 (RH 2212), Johnson and Sneath J34, KCTC 12681, Lautrop AB1182, LMG 995, LMG 1235, LMG 10630, LUH 1717, NCTC 10304, NIPH 1860, RUH 2218, **Stenzel and Mannheim P544/60**, Torry 7-37, USCC 1603	≤1962	Germany	Conjunctiva	Stenzel
*Acinetobacter indicus*					
84	ATCC 17976, Baumann 98, Bouvet and Grimont 20, CDC KC769 (NCDC KC769), CIP 53.82, LMG 1023, **Piéchaud strain Faucon**, Piéchaud 5382	≤1951	France	Post-operative meningitis	Piéchaud
*Acinetobacter higginsii* **=Genospecies 16BJ**					
85	ANC 4585, **CIP 56.2**, CNCTC 8062	1955	France	Liver abscess	Second
86	ATCC 17988, Baumann 34, BCRC 15883, Bouvet and Grimont 78, CCM 9243^T^, CCUG 996^T^, CDC KC727, CIP 70.18^T^, CNCTC 7558^T^, **Henriksen 4233/62**, Juni 40, Johnson and Sneath J26, LMG 1031^T^, LUH 1731, NIPH 1872^T^, Sahl TG19627	1962	Unknown	Urine	S.D. Henriksen
*Acinetobacter baylyi*					
87	ATCC 33304, BD4, **BD-4**, CCM 2881, CNCTC 7795, DSM 586, Juni 1, NCAIM B.01388, NIPH 2312, Sahl TG19579	1961	Unknown	Soil	Juni
88	**ADP1**, BD413, MDayP1, NCIMB 11826, VTT E-031437	1961	Unknown	Mutant of BD4 (1969)	Day
*Acinetobacter* sp.					
89	CCUG 3356, **DB264**, Johnson and Sneath J10, LMG 1053, NCDO 494, NCIMB 8596 (NCIB 8596), Thornley 28	1931	Unknown	Ropy milk	A.G. Lochhead
90	**NCIMB 5178**	≤1939	Unknown	Unknown	F.C. Happold
91	ATCC 9956, **Deacon 5-156A**, Hugh 484 (RH 484)	≤1945	Italy	Clinical sample	W.E. Deacon
92	**NCTC 7250**, strain Bellairs	≤1947	UK	Cerebrospinal fluid	H.W. Dalton
93	**NCTC 7363**, strain Poultney	≤1947	UK	Unknown	A.P. Peeney
94	**NCTC 7461**, strain Crook	≤1948	UK	Tuberculosis sinus	C.H. Lack
95	**NCTC 7462**, strain Hourigan	≤1948	UK	Tuberculosis sinus	A.P. Peeney
96	ATCC 19678, Hugh 2207 (RH 2207), **Schaub 77**	≤1948	USA	Urine	I.G. Schaub
97	ATCC 19569, Canad-226 90, Hugh 2316 (RH 2316), LMG 1040, NCIMB 9213 (NCIB 9213), Ontario B10, **Schaub 90**, Sneath D.410, Thornley 35	≤1948	USA	Urine	I.G. Schaub
98	ATCC 19682, Hugh 2314 (RH 2314), LMG 1042, NCCB 82033 (LMD 82.33), NCIMB 9211 (NCIB 9211), Ontario B7, **Schaub strain Eddy**, Sneath D.408, Thornley 33	Around 1948	Unknown	Unknown	I.G. Schaub
99	ATCC 19681, **Ferguson B5W 81**, Hugh 2228 (RH 2228), Stuart H1167	≤ 1950	Unknown	Unknown	I.G. Schaub
100	ATCC 19679, **Ferguson B5W 69**, Hugh 2225 (RH 2225), Stuart H1168	≤1950	Unknown	Unknown	I.G. Schaub
101	ATCC 19680, **Ferguson B5W 79**, Hugh 2227 (RH 2227), Stuart H1169	≤1950	Unknown	Unknown	I.G. Schaub
102	ATCC 27230, Billing BT10, **Ferguson B5W 99**, Juni 54, LMG 1063, NCIMB 9301 (NCIB 9301), Ontario B25, Stuart W83 (or W832), Thornley 39	≤1950	Unknown	Unknown	C.A. Stuart
103	ATCC 27224, Billing BT1, Cherry B5W 2, **Ferguson B5W 2**, LMG 1059, NCIMB 9292 (NCIB 9292)	≤1950	Unknown	Urine	W.W. Ferguson
104	ATCC 27225, Billing BT3, **Ferguson B5W 4**, Juni 53, NCIMB 9294 (NCIB 9294)	≤1950	Unknown	Sputum	W.W. Ferguson
105	ATCC 27226, Billing BT4, **Ferguson B5W 6**, LMG 1060, NCIMB 9295 (NCIB 9295)	≤1950	Unknown	Urine	W.W. Ferguson
106	ATCC 27227, Billing BT5, **Ferguson B5W 13**, LMG 1061, NCIMB 9296 (NCIB 9296)	≤1950	Unknown	Nasopharynx	W.W. Ferguson
107	ATCC 27228, Billing BT6, **Ferguson B5W 19**, LMG 1062, NCIMB 9297 (NCIB 9297)	≤1950	Unknown	Urine	W.W. Ferguson
108	ATCC 27229, Billing BT7, **Ferguson B5W 53**, NCIMB 9298 (NCIB 9298)	≤1950	Unknown	Blood	W.W. Ferguson
109	ATCC 27237, **1043**	≤1952	USA	Faeces	E.O. King
110	ATCC 17912, Baumann 38, **Billing A1;ST1**, BCRC 15887 (CCRC 15887), CDC KC714, CIP 70.13, Hugh 461 (RH 461), NCIMB 9115 (NCIB 9115), NCTC 8698	1952	Unknown	Unknown	Billing
111	ATCC 14682, CDC KC138, CNCTC 6754, CNCTC Mi 7/65, Hugh 2204 (RH2204), **Seeliger V 2240-52**	≤1953	Unknown	Spinal fluid	Seeliger
112	82 Groombridge, Alexander 63, **NCTC 9427**	≤1954	UK	Meningitis	Knox
113	ATCC 19649, **Billing B4**, Hugh 463 (RH 463)	≤1955	Unknown	Unknown	Billing
114	**Brisou 66**, Johnson and Sneath J12, LMG 1054, NCIMB 9021 (NCIB 9021)	≤1957	Unknown	Unknown	Brisou
115	**NCDO 1092**, NCIMB 701092	1957	Unknown	Cow’s teat	K. Neave
116	**24**, 24(M6), ATCC 14292, Baumann 27	≤1958	USA	Human	S.G. Cary
117	**44**, 44(M3), ATCC 14291, Baumann 26	≤1958	USA	Human	S.G. Cary
118	ATCC 14290, Baumann 66, **CDC 1847**, Hugh 2418 (RH 2418), Johnson and Sneath J24	≤1958	USA	Urine	S.G. Cary
119	ATCC 14294, **CDC 1856**, CNCTC 6753, CNCTC Mi 6/65, Hugh 2422 (RH 2422)	≤1958	USA	Throat	S.G. Cary
120	Enjalbert 4.589, **Kambou 4.589**, LMG 1058, NCCB 82034 (LMD 82.34), NCIMB 9284 (NCIB 9284)	≤1959	Unknown	Unknown	Enjalbert
121	Enjalbert 88.860, **Kambou 88.860**, NCCB 82035 (LMD 82.35), NCIMB 9287 (NCIB 9287)	≤1959	Unknown	Unknown	Enjalbert
122	Enjalbert 87.059, **Kambou 87.059**, LMG 1065, NCIMB 9303 (NCIB 9303)	≤1959	Unknown	Unknown	Enjalbert
123	**Kambou 88.080**, LMG 1066, NCIMB 9304 (NCIB 9304)	≤1959	Unknown	Unknown	Enjalbert
124	**Kambou 88.125**, NCIMB 9305 (NCIB 9305)	≤1959	Unknown	Unknown	Enjalbert
125	ATCC 13809, BCRC 12288 (CCRC 12288), Dijkshoorn AC53, Dijkshoorn 76, IAM 12580, IFO 12147, JCM 20657, **Kinney 14** A2, NCCB 71043 (LMD 71.43), NCIMB 9542 (NCIB 9542)	1960	Unknown	Soil	R.W. Kinney
126	ATCC 15567, DSM 30009, **Ellis 1972**, Ellis 5209, Hugh 1935 (RH 1935)	≤1961	Unknown	Chicken	E.M. Ellis
127	ATCC 15566, **Ellis 2409**, Ellis 5345, Hugh 1936 (RH 1936)	≤1961	Unknown	Bovine	E.M. Ellis
128	ATCC 27238, **7186**	≤1961	USA	Unknown	E.O. King
129	31BK, ATCC 27231, **Kass 61-29261**	1961–62	USA	Blood (bacteraemia)	E.H. Kass
130	131BK, ATCC 27233, **Kass 1170**	1961–62	USA	Blood	E.H. Kass
131	154BK, ATCC 27235, Juni 55, **Kass 50372**	1961–62	USA	Blood	E.H. Kass
132	162BK, ATCC 27236, **Kass 54651**	1961–62	USA	Infected cut lesion	E.H. Kass
133	**Lautrop A-4**, LMG 1161, NCTC 10290	≤1962	Denmark	Skin abscess	Lautrop
134	**Lautrop A-15**, NCTC 10291	≤1962	Denmark	Infant’s liver	Lautrop
135	ATCC 27234, **4935**	≤1962	Unknown	Blood	W.H. Ewing
136	ATCC 27232, **4936**	≤1962	Unknown	Blood	W.H. Ewing
137	ATCC 27241, **8647**	≤1962	USA	Urine	W.B. Cherry
138	ATCC 27248, **8870N**	≤1963	Israel	Throat of well-baby	W.B. Cherry
139	ATCC 27239, **8886N**	≤1963	USA	Cerebrospinal fluid	W.B. Cherry
140	ATCC 27245, **9346**	≤1963	USA	Ear	W.B. Cherry
141	ATCC 27240, **9358**	≤1963	USA	Blood	W.B. Cherry
142	ATCC 27243, **9378**	≤1963	USA	Bronchus	W.B. Cherry
143	ATCC 27242, **9391**, Juni 60	≤1963	USA	Sputum (pneumonia)	W.B. Cherry
144	ATCC 27246, **9469** N	≤1963	USA	Feet	W.B. Cherry
145	Baumann 17, CCUG 2878, **NCIMB 9689** (NCIB 9689)	≤1964	Unknown	Soil	Walker
146	ATCC 19683, Hugh 2272 (RH 2272), **King 7411**, Nelson N1140, Nelson 7	≤1965	Unknown	Blood	E.O. King
147	ATCC 19684, Hugh 2273 (RH 2273), **King 6801**, Nelson N1141, Nelson 8	≤1965	Unknown	Autopsy	E.O. King
148	**ATCC 11959**, Hugh 485 (RH 485)	≤1966	Unknown	Soil	J.P. Gilman
149	ATCC 27247, **9092N**	≤1966	USA	Ankle abscess	W.B. Cherry
150	ATCC 27249, **47-8628**	1966	USA	Urine	W.B. Cherry
151	ATCC 27250, **47-8643**	1966	USA	Ear	W.B. Cherry
152	ATCC 27251, **47-8928**	1966	USA	Pneumonia	W.B. Cherry
153	ATCC 27252, **47-8922**	1966	USA	Pus	W.B. Cherry
154	ATCC 27253, **47-9192**	1966	USA	Throat	W.B. Cherry
155	ATCC 27254, **47-9253**	1966	USA	Urine	W.B. Cherry
156	ATCC 23237, CIP 68.40, DSM 30013, **Klinge E2241/60**, LMG 1051, Thornley 45	≤1967	Germany	Human source	Legler
157	CECT 8149, DSM 25761, NCIMB 10764, **Thornley MJT/F5/122**, WDCM 00060	≤1967	Unknown	Poultry carcass	M.J. Thornley
158	NCCB 88033 (LMD 88.33), **Thornley MJT/F5/158**	≤1967	Unknown	Poultry carcass	LMD
159	ATCC 10153, **FDA PCI-3**, Hugh 24 (RH 24)	≤1967	Unknown	Unknown	FDA
160	ATCC 15473, CNCTC 6755, CNCTC Mi 9/66, **FDA PCI 1788**, Hugh 2287 (RH 2287)	≤1968	USA	Urine	T.W. Chang
161	**Nakasuji A43**, NBRC 13006 (IFO 13006)	≤1969	Unknown	Unknown	Nakasuji
**Unclassified**					
162	**ATCC 12448**, L 35 FTS	Unknown	Unknown	Unknown	S.R. Venkataraman

*ATCC, American Type Culture Collection; Baumann and Doudoroff, reference no. [2]; BCRC, Bioresource Collection and Research Center (Taiwan); Bouvet and Grimont, reference no. [22]; CAIM, Collection of Aquacultural Important Microorganisms (Mexico); CCT, Coleção de Culturas Tropical (Brazil); CCUG, Culture Collection University of Gothenburg (Sweden); CECT, Spanish Type Culture Collection; CIP, Collection de l’Institut Pasteur (France); CNCTC, Czech National Collection of Type Cultures; Delft, Delft School of Microbiology (the Netherlands); Beijerinck, reference no. [21]; LMD, Laboratorium voor Microbiologie Delft (transferred to NCCB, the Netherlands); Dijkshoorn [71,78], reference DOI: 10.1099/00222615-23-4-313; Dijkshoorn serial no., reference no. [29]; DSMZ, Deutsche Sammlung von Mikroorganismen und Zellkulturen (German Collection of Microorganisms and Cell Cultures); FIRDI, Food Industry Research and Development Institute (Taiwan, transferred to BCRC); IAM, Institute of Applied Microbiology, University of Tokyo (Japan) (transferred to JCM); JCM, Japan Collection of Microorganisms; Johnson and Sneath, reference no. [130]; Juni, reference no. [86]; KCTC, Korean Collection for Type Cultures; LMG, Laboratorium voor Microbiologie Universiteit Gent (Belgium); NBIMCC, National Bank for Industrial Microorganisms and Cell Cultures (Bulgaria); NBRC, NITE Biological Resource Center (Japan); IFO, Institute for Fermentation (Japan) (transferred to NBRC); NCCB, Netherlands Culture Collection of Bacteria; NCIMB, National Collection of Industrial, Food and Marine Bacteria (UK); NCIB, National Collection of Industrial Bacteria (UK); NCTC, National Collection of Type Cultures (UK); NIPH, National Institute of Public Health, strain designation used by Alexander Nemec (Czech Republic); NRRL, the Agricultural Research Service Culture Collection (USA); NZRM, New Zealand Reference Culture Collection Medical Section; RUH, strain designation used by Lenie Dijkshoorn (The Netherlands), reference doi: 10.1099/00207713-47-4-1179; VTT, Valtion teknillinen tutkimuskeskus (Technical Research Centre of Finland); Hugh, reference no. [85]; RH, Reese and Hugh (reference no. [85]); Sahl, reference no. [101]; CCM, Czech Collection of Microorganisms; CDC (NCDC), Centers for Disease Control and Prevention (USA); Kallio, reference nos. [145] and [146]; NCAIM, National Collection of Agricultural and Industrial Microorganisms (Hungary); NCIM, National Collection of Industrial Microorganisms (India); Allen, reference no. [78]; Dijkshoorn AC, strain designation used by L. Dijkshoorn (The Netherlands); NCDO, National Collection of Dairy Organisms (UK); Thornley, reference no. [93]; Deacon, reference no. [84]; CCRC, Culture Collection and Research Centre (Taiwan); Schaub, reference nos. [92] or [94]; Canad-226, Ontario Department of Health Laboratory, Toronto (Canada) (see reference no. [72]); Ontario, Ontario Department of Health Laboratory, Toronto (Canada) (see reference no. [93]); Sneath, reference no. [72]; CCEB, Culture Collection of Entomophagous Bacteria (Czech Republic); Ferguson, reference no. [100]; Schaub and Hauber, reference nos. [92] or [94]; Lampe, A. S. Lampe (according to NCCB); IMI, International Mycological Institute, transferred to CABI culture collection (UK); USCC, University of Surrey Culture Collection (UK); Lautrop, reference no. [154]; Stuart, reference no. [97]; Billing, reference no. [144]; Piéchaud, reference nos. [105] and [108]; Seeliger, reference no. [137]; Courtieu reference no. [128]; Enjalbert, reference no. [119]; Kambou, reference no. [153]; Stenzel and Mannheim, reference nos. [120] and [138]; Torry, Torry Research Station (Scotland); Kass, reference no. [166]; Brisou, reference no. [44]; MTCC, Microbial Type Culture Collection and Gene Bank (India); Rubinsten, reference nos. [90] and [91]; Audureau, reference no. [36]; HAMBI, Microbial Domain Biological Resource Centre (Finland); ANC, strain designation used by the Centre for Epidemiology and Microbiology at the National Institute of Public Health (Czech Republic); Lwoff, reference no. [34]; Pintér and Bende, reference no. [133]; LUH, strain designation used by Lenie Dijkshoorn (The Netherlands), reference doi: 10.1099/00207713-47-4-1179; Klinge, reference no. [126]; Henriksen, reference nos. [5,33] and [129]; Ellis, reference no. [160]; King, reference no. [85]; Nelson, reference no. [167]; Nakasuji, reference no. [175]; WDCM, World Data Centre for Microorganisims; FDA, Food and Drug Administration (USA). Strain designations used in this manuscript were made bold. T, Type strain.

Delft L360 is another isolate of Beijerinck’s ‘*M. calco-aceticus*’ bacteria that is still preserved until now (Table 1, 2). Nonetheless, we found intermixes between the culture deposits of Delft 1 and Delft L360. For instance, LMG 1046^T^ was linked to both ‘Delft 1’ and ‘Delft L360’ [25]. The genetic similarity between DSM 30006^T^ (GenBank: BBTM00000000.1), a subculture of Delft 1 and Sahl TG19588 (GenBank: AMIX00000000.1), a subculture of Delft L360, was also high as both of them belonged to sequence type (ST) 62, according to the multilocus sequence typing Pasteur scheme [27], and both carried the chromosomal gene alleles *bla*_OXA213_ and *bla*_ADC-127_ (Table S2). The colonies of ATCC 19638 [28] and NCIMB 9029 [24], both sub-cultured from Delft L360, were described as opaque, suggesting that Delft L360 could be a sub-culture of an opaque colony of Delft 1. Alternatively, Delft L360 and Delft 1 might be independent isolates of the same strain.

Another isolate of Beijerinck’s ‘*M. calco-aceticus*’ is still preserved as Delft L361 (Table 1, 3). This isolate was reclassified as * A. calcoaceticus* by DNA–DNA hybridization, ribotyping and repetitive extragenic palindromic sequence-based PCR typing [29,31]. LMD 22.17 (Delft L361) and ATCC 23055^T^ (Delft 1) had the same *EcoRI* ribotype [32], suggesting that they are closely related. However, the exact relationship between these isolates is not known since the genome of Delft L361 is not available.

## The genus *Moraxella*

In 1939, Lwoff proposed a novel genus to separate two species, ‘*Haemophilus lacunata*’ and ‘*Haemophilus duplex*’, from the tribe ‘*Haemophileae*’ since they neither resembled the morphology of ‘*Haemophileae*’ nor required haematin or phosphopyridinenucleotide as growth factors (Fig. 1) [33,34]. The novel genus was named *Moraxella*, after the Swiss ophthalmologist Victor Morax, and consisted of *Moraxella lacunata*, the type species of the genus, and ‘*Moraxella duplex*’ (Fig. S1). The speciation was mainly based on the observation that *M. lacunata* required the addition of serum to grow, while ‘*M. duplex*’ could grow in peptone water alone [34]. Based on their phenotypic and growth features, ‘*M. duplex*’ was divided into three varieties, i.e. ‘*M. duplex* var. *liquefaciens*’ (able to liquefy gelatin and coagulated serum, which was also a trait of *M. lacunata*), ‘*M. duplex* var. *nonliquefaciens*’ (unable to liquefy gelatin or coagulated serum) and ‘*M. duplex* var. *josephi*’ (unable to liquefy gelatin or coagulated serum; distinguished by being Gram-positive), plus one conditional variety, ‘*M. duplex* des Bovidés’, which required further validation [34].

Later on, and in contrast to the other *Moraxella* species and varieties, strains of *Moraxella bovis* (‘*M. duplex* des Bovidés’) were reported to be haemolytic [33,35]. In 1940, Audureau divided the species *M. lacunata* into two varieties, ‘*M. lacunata* var. *typica*’ (able to liquefy gelatin and coagulated serum) and ‘*M. lacunata* var. *atypica*’ (unable to liquefy gelatin or coagulated serum) (Fig. 1) [36]. Audureau also proposed the addition of a novel species, ‘*Moraxella lwoffi*’, to separate strains that were able to grow in a simple synthetic medium without growth factors. ‘*Moraxella lwoffi*’ was divided into two varieties, ‘*Moraxella lwoffi* var. *bacteroides*’ (normal forms) and ‘*Moraxella lwoffi* var *brevis*’ (short forms) [36]. The author also reported that ‘*Moraxella lwoffi* var. *bacteroides*’ did not grow at 39 °C, while ‘*Moraxella lwoffi* var *brevis*’ did.

## From *Moraxella* to *Acinetobacter lwoffii* and *Acinetobacter johnsonii*

Four of Lwoff and Audureau’s *Moraxella* isolates that were cultured in or before 1940 are available today. Three of them, Lwoff 1, Lwoff 2 and Audureau strain brevis, are now reclassified as *A. lwoffii* based on DNA–DNA hybridization and whole genome sequence data (GenBank: CP118963.1 to CP118968.1 for DSM 2403^T^, a subculture of Lwoff 1; UFSE00000000.1 for NCTC 5867, a subculture of Lwoff 2; and APOG00000000.1 for CIP A162, a subculture of Audureau strain brevis; 37, 38). Lwoff 1 (Table 1, 65) was earlier described as ‘*Moraxella lwoffi* var. *brevis*’ or ‘*Moraxella lwoffi* var. *bacteroides*’ [25] but has become the type strain of *A. lwoffii* since 1964 [22,37, 38]. Similarly, Lwoff 2 (Table 1, 66) was first described as ‘*M. lacunata* var. *atypical*’, ‘*Moraxella lwoffi* var. *brevis*’, ‘*Moraxella lwoffi* var. *nonliquefaciens*’ (see below) or ‘*A. calcoaceticus* var. *Iwoffi*’ [25,39, 40], while it is now one of the reference strains for *A. lwoffii* [41,43]. The classification of Audureau strain brevis (Table 1, 67) was changed from ‘*Moraxella lwoffi* var. *brevis*’ and ‘*Moraxella lwoffi* var. *nonliquefaciens*’ to genospecies 9 [22], which is currently named *A. lwoffii* (GenBank: APOG00000000.1, 8, 40).

The fourth isolate, Audureau strain bacteroides (Table 1, 57), was related to a case of chronic conjunctivitis [36]. It was first described as ‘*Moraxella lwoffi* var. *bacteroides*’ or ‘*Moraxella lwoffi* var. *nonliquefaciens*’ [25,39] and later assigned to genospecies 7, corresponding to *A. johnsonii* [22]. It is worth mentioning that the whole genome of ATCC 17969, a subculture from Audureau strain bacteroides, is available at GenBank (BioSample: SAMN09914435, SRA: SRP159200), while the isolate itself cannot be found online at the American Type Culture Collection (ATCC) website.

## From *A. lwoffii* to *Acinetobacter pseudolwoffii*

Isolate CIP 64.10 (Table 1, 63), assumed to be a subculture of Lwoff 1 via NCTC 5866^T^, was found to be closely related to Brisou isolate 65 (Table 1, 64), isolated before 1958 [44], based on their DNA–DNA homology [22]. The two isolates were grouped into genospecies 8, which was found to be closely related to genospecies 9 [22]. However, the authenticity of CIP 64.10 was later questioned by Tjernberg and Ursing and by Touchon *et al*. who found that the average nucleotide identity value between the genome sequences of CIP 64.10 and NCTC 5866^T^ was as low as 88.3% [8,42]. In 2019, Nemec *et al*. confirmed that these two isolates are different and demonstrated that they belong to two phylogenetically related but taxonomically distinct species [38].

The name *A. lwoffii* was assigned to NCTC 5866^T^ and the other isolates of Bouvet and Grimont genospecies 9, based on the emended description of this species, while the name *A. pseudolwoffii* sp. nov. was proposed for Bouvet and Grimont genospecies 8 represented by CIP 64.10 and Brisou isolate 65 [38]. The origin, date of isolation and history of CIP 64.10 are unknown. CIP 64.10 is currently not available from the Collection de l’Institut Pasteur but is deposited at the Czech National Collection of Type Cultures as CNCTC 7645.

## From Vibrio 01 to *Acinetobacter guillouiae*

In 1932, Happold and Key described a Gram-negative bacterium, *Vibrio* strain 01, that was capable of degrading monohydric phenols [45]. This strain was later included in several studies investigating the metabolism of aromatic compounds [46,47]. The strain was deposited by W. C. Evans in the National Collection of Industrial Bacteria (NCIB, Torry Research Station, Aberdeen, Scotland) in 1950 as NCIB 8250 (Table 1, 81) and since then NCIB 8250 has commonly been referred to as Vibrio 01 [2,43, 48]. However, it soon appeared that NCIB 8250 had different properties from those of the original strain of Vibrio 01, which was most likely lost [49].

NCIB 8250 was then described as ‘*Moraxella lwoffi*’ [50,51]. It was later reclassified into the ‘*Acinetobacter-Moraxella*’ group [52] and finally assigned as the type strain of *A. guillouiae* [53]. Notably, the amikacin resistance gene *aphA6* was present in the genome of a subculture of NCIB 8250^T^ (CIP 63.46^T^; GenBank: APOS00000000.1). PCR screening and antimicrobial susceptibility testing identified *A. guillouiae* as a silent reservoir of *aphA6* [54]. By searching the records of the UK National Collection of Industrial, Food and Marine Bacteria (NCIMB) collection, we found another isolate, NCIMB 5178 (Table 1, 90), deposited by Happold in 1939. Later, NCIMB 5178 was subjectively identified as *A. calcoaceticus* [55], reflecting the undeveloped species classification of the genus *Acinetobacter* at that time. Thus, the question of the species affiliation of NCIMB 5178 remains open.

## The genus *Alcaligenes*

The name ‘*Bacillus faecalis alcaligenes*’ was first used by Petruschky in 1896 to describe a peritrichous motile rod of intestinal origin that was non-spore-forming, did not produce acid in sugar broths and caused alkalinity in milk [56,57]. The generic name *Bacillus* was changed to *Bacterium* and a binomial form of the name, ‘*Bacterium alcaligenes*’, was proposed by Mez in 1898 [58,59]. The genus *Alcaligenes* was formally coined in 1918, grouping a variety of organisms characterized by their general lack of fermentative power (not fermenting glucose or lactose) and their ability to increase alkalinity in milk and in culture media [60]. Nine species of the genus *Alcaligines* were listed in the first edition of Bergey’s Manual of Determinative Bacteriology [61], including *Alcaligines fecalis* (motile, do not liquefy gelatin), ‘*Alcaligines metalcaligines*’ (non-motile, did not liquefy gelatin) and ‘*Alcaligines marshallii*’ (non-motile, liquefy gelatin). Notably, ‘*Alcaligines metalcaligines*’, representing a later name of ‘*Bacillus metalcaligines*’ [62,63], was described as Gram-positive in this edition [61]. ‘*Alcaligenes marshallii*’ was linked to a slimy milk organism known as ‘*Bacillus* B of Marshall’ [64,66]. This organism was first assigned the trinomial name ‘*Bacterium lactis marshalli*’ [64] and later the binomial name ‘*Bacterium marshalli*’ [67].

Another species, ‘*Alcaligenes viscosus*’, was described in the sixth edition of Bergey’s Manual based on its ability to produce ropiness in milk [35]. ‘*Alcaligenes viscosus*’ did not liquify gelatin and was described as motile. This species was then named ‘*Alcaligenes viscolactis*’ in the seventh edition of Bergey’s Manual of Determinative Bacteriology [68], where it was redescribed as non-motile. We also noted that the description of ‘*Alcaligenes metalcaligenes*’ was changed to Gram-negative in these two editions. The name ‘*Alcaligenes viscolactis*’ can be traced back to an organism isolated from ropy milk in 1889 and first named ‘*Bacillus lactis viscosus*’ [62,63]. The name was then changed to ‘*Bacterium lactis viscosum*’ [59,64, 69], ‘*Bacterium viscolactis*’ [58], ‘*Alcaligenes viscosum*’ [70] and ‘*Lactobacillus viscosus*’ [61] and back to ‘*Alcaligenes viscosus*’ [56].

In 1937, Henriksen cultured a number of Gram-negative coccobacilli isolates from human respiratory tract samples [71]. The isolates haemolysed rabbit blood and were therefore named ‘*Alcaligenes haemolysans*’. One of Henriksen’s original cultures was preserved in the UK National Collection of Type Cultures as NCTC 5043 [72]. Unfortunately, this isolate was later discarded [73], and all of Henriksen’s isolates from his 1937 study were also lost [5]. In 1954, the names ‘*Acinetobacter marshalli*’ and ‘*Acinetobacter viscosum*’ were proposed by Brisou and Prévot [16]. However, Baumann *et al*. did not use these two epithets for the corresponding isolates [2].

## From ‘*Alcaligenes viscosus*’ to *A. johnsonii*

We found an *Acinetobacter* strain, ATCC 9036 (Table 1, 56), deposited by B. W. Hammer in the ATCC under the name ‘*Alcaligenes viscosus*’. This isolate was most likely part of a study that was published in 1936 [74]. ATCC 9036 was classified in genospecies 7, corresponding to *A. johnsonii* [22], and this classification was later confirmed by whole-genome sequencing (GenBank: BJUJ00000000.1). Another strain, DB264 (Table 1, 89), was collected by A. G. Lochhead in 1931 and was deposited first in the Division of Bacteriology and Dairy Research, Ottawa, and then in the UK National Collection of Dairy Organisms (NCDO), now part of the NCIMB. Both ATCC 9036 (NCDO 715) and DB264 (NCDO 494) were unable to liquefy gelatin or haemolyse blood [75]. However, we could not find any further taxonomic studies related to DB264. Interestingly, strain ATCC 12448 (Table 1, 162) was deposited by S. R. Venkataraman under the name ‘*Achromobacter viscosus* Sreenivasan’ [28]. The classification of ATCC 12448 was probably done based on the second edition of Bergey’s Manual [76], and this isolate should be considered as ‘*Alcaligenes viscosus*’ (or ‘*viscolactis*’), according to the sixth and seventh editions [77]. Genome analysis is needed to determine whether ATCC 12448 is a member of the genus *Acinetobacter*.

## The genus *Achromobacter*

A novel tribe, ‘*Achromobactereae*’ trib. nov., was proposed in the first edition of Bergey’s Manual of Determinative Bacteriology in 1923 to describe Gram-negative rods that sized small to medium, were motile or non-motile, occurred principally in water and soil and did not form pigment on agar or gelatin but could produce a brownish growth on potato (Fig. 1) [61]. The tribe included only a single genus, named *Achromobacter*, with 51 species. ‘*Achromobacter lacticum*’ was one of the non-motile species that were unable to liquefy gelatin [61]. It was able to acidify milk but unable to reduce nitrates or to form indole. ‘*Achromobacter venosum*’ referred to motile rods with peritrichous flagella that were unable to liquefy gelatin, to reduce nitrates or to form indole [61]. ‘*Achromobacter delictatulum*’ was motile with peritrichous flagella, able to liquefy gelatin, acidify milk and reduce nitrates, but unable to form indole [35,61].

## The earliest two isolates of *Acinetobacter baumannii*

In 1944, Allen *et al*. reported the isolation of bacteria from samples of retting shrubs, which were capable of clearing emulsions of guayule resins [78]. Three nonmotile, Gram-negative, short, coccoid rod isolates (4H, 6H and 13H) were classified as ‘*Achromobacter lacticum*’ based on their phenotypes. Analysis of the whole genomes of 4H (GenBank: JJOC00000000.2) and 6H (GenBank: CP028138.1 and CP028139.1) reassigned them into *Acinetobacter baumannii* [79,80]. To our knowledge, 4H and 6H are probably the oldest accessible isolates of *A. baumannii* (Table 1, 6 and 7). Inconsistently, NCIB 8209 (sub-cultured from Allen 6H) was later reported to be motile and to have peritrichous flagella [81].

## The tribe ‘*Mimeae*’

In 1942, G. G. De Bord assigned a group of clinical isolates obtained from cases of conjunctivitis and vaginitis to a novel tribe, ‘*Mimeae*’ trib. nov., based on the following common features: (1) pleomorphism with the diplococcal form predominating on the solid media and a more nearly even distribution of cocci, rods and filaments in liquid media; (2) a modified bipolar staining; (3) Gram negativity with some retention of Gram positivity; (4) encapsulation; and (5) the physical appearance of the colonies on various media (Fig. 1) [82]. The name referred to their morphological resemblance to members of the genus *Neisseria*, a notable characteristic of these isolates. The tribe included three genera, ‘*Mima*’ gen. nov. (described as not fermenting carbohydrates), ‘*Herellea*’ gen. nov. (fermenting certain carbohydrates with production of acid only) and ‘*Colloides*’ gen. nov. (fermenting certain carbohydrates with production of acid and gas). ‘*Mima polymorpha*’, including the variety ‘*oxidans*’, was the only species described by De Bord in the genus ‘*Mima*’ [33,82, 83]. The genus ‘*Herellea*’ also included only one species, named ‘*Herellea vaginicola*’.

## Deacon’s three isolates: from ‘*Herellea*’ to *A. baumannii* and from ‘*M. polymorpha’* to *A. lwoffii*

Based on De Bord’s description, Wilbur E. Deacon classified ten and eight of his clinical bacterial cultures into the genus ‘*Herellea*’ and the genus ‘*Mima*’, respectively [84]. Three of these isolates (two ‘*Herellea*’ and one ‘*Mima*’) are currently reclassified as *Acinetobacter*. The first ‘*Herellea*’ isolate, Deacon 6-561 (Table 1, 8), was reclassified as *A. baumannii* based on the results of DNA–DNA homology studies [2,22, 43, 85, 86]. The identification was confirmed by comparative analysis of partial sequences of the *rpoB* gene and its flanking spacers [87]. The second ‘*Herellea*’ isolate, Deacon 5-156A (Table 1, 91), was described as * A. calcoaceticus* in 1973 [88], but also as *A. baumannii* [89], although both designations could be incorrect, as we could not find a conclusive classification for this isolate in any publication or GenBank record. Deacon 5-156A is currently deposited at ATCC as *Acinetobacter* sp. [28]. The third isolate, Deacon H-B (Table 1, 68), was first described as ‘*M. polymorpha*’ or ‘*Moraxella lwoffi* var. *nonliquefaciens*’ (see below) and then renamed as *A. lwoffii* based on DNA–DNA homology and genome sequencing (GenBank: APQT00000000.1) [2,22, 43].

## From *Alcaligenes fecalis* to *A. lwoffii* and *Acinetobacter junii*

Three isolates collected in 1947 from human faeces (Rubinsten strain 1), spinal fluid (Rubinsten strain 18), and blood (Rubinsten strain 19) were initially described as *Alcaligenes fecalis* (Table 1, 50, 69 and 70) [90,91]. These isolates were later reclassified as * A. junii* (Rubinsten strain 1) and *A. lwoffii* (Rubinsten strains 18 and 19) based on their 16S rRNA gene sequence and matrix-assisted laser desorption/ionization-time of flight (MALDI-TOF) MS results [39], although neither these sequences nor the MALDI-TOF results are publicly available. The motility of these isolates should be reassessed since *Alcaligenes fecalis* was recognized to be motile.

## From ‘*Bacterium anitratum*’ to *A. baumannii*

In 1948, Schaub and Hauber proposed the name ‘*B. anitratum*’ sp. nov. for a taxonomically new and biochemically and serologically homogeneous group of 15 clinical isolates (Fig. 1) [92]. The isolates were placed in the genus ‘*Bacterium*’, as this genus was reserved for organisms not yet assigned to any of the recognized genera, and they were given the species epithet ‘*anitratum*’ since they did not reduce nitrates, a feature that distinguished them from the majority of Gram-negative bacilli, particularly the *Enterobacteriaceae*. Four of these isolates (nos. 77, 81, 90 and 93, all clinical urine isolates) were later assigned to the genus *Acinetobacter*. Based on DNA–DNA homology, partial *rpoB* sequence analysis and whole-genome sequencing, isolate 81 (Table 1, 12) belonged to *A. baumannii* (GenBank: CP045110.1, CP045108.1 and CP045109.1) [22,23, 87], as well as isolate 93 (Table 1, 13), according to the whole-genome assembly of ATCC 19187 [28]. Isolate 90 (Table 1, 97) could also be correlated to *A. baumannii* as it was described as phenon 2 by Thornley in 1967 although this needs to be validated (see below) [93]. We could not find any further taxonomic information about isolate 77 (Table 1, 96), other than its basic phenotypic classification in 1948 and 1968 [85,92]. Both isolates 77 and 90 are currently described as *Acinetobacter* sp., awaiting further classification.

Another isolate, Schaub Biol. 1 (Table 1, 14), was found in literature and in the records of several national culture collections. It is worth mentioning that Schaub Biol. 1 was not one of the 15 isolates mentioned in the previous paragraph [94]. In 1964, Steel and Cowan proposed the designation of Schaub Biol. 1 as the type strain for ‘*B. anitratum*’ (see below) [37]. However, since Schaub Biol. 1 was not part of the original description of ‘*B. anitratum*’, Hugh and Reese nominated isolate 81 as the type for ‘*B. anitratum*’ instead of Schaub Biol. 1 [94]. The whole-genome sequence analysis of CCUG 33549, a subculture of Schaub Biol. 1, supported its identification as *A. baumannii* (GenBank: JAMOLD000000000.1). Interestingly, CCUG 33549 showed high similarity to * A. baumannii* ATCC 19606^T^ (a subculture of Schaub isolate 81), and ATCC 19187 (a subculture of Schaub isolate 93) as all of them belonged to ST52 (MLST Pasteur scheme) [27] and ST931 (MLST Oxford scheme) [95], and both CCUG 33549 and ATCC 19606^T^ had the intrinsic genes *bla*_OXA-98_ and *bla*_ADC-158_ and the acquired sulphonamide resistance gene *sul2* [96]. These results suggest that Schaub Biol. 1, isolate 81 and isolate 93 may all come from the same strain.

## From B5W to *Acinetobacter nosocomialis* and *A. baumannii*

In 1949, Stuart *et al*. described the morphological and biochemical characteristics of 81 isolates that were classified under a novel bacterial group name, B5W (Fig. 1) [97]. B5W was used to replace an older designation, ‘*Bacillus* P’, which described a number of cultures of an unknown organism [98,99]. B5W, corresponding to a white colony isolated from the fifth specimen from institution B, was probably selected for lack of a species name [100]. One of Stuart’s isolates, A267 (Table 1, 82), was later identified as *A. nosocomialis* based on DNA–DNA homology and genome sequence analysis (GenBank: CP029351.1) [2,22, 43]. A267 is probably the oldest available *A. nosocomialis*, isolated about 40 years before the type strain of this species (RUH 2376, LMG 10619^T^, CCM 7791^T^, NIPH 2119^T^, DSM 102856^T^ and CNCTC 7433^T^) [23,101].

We found three other isolates, Schaub Biol. 2 (Table 1, 15), Schaub strain Eddy (Table 1, 98) and Stuart B5W 3777 (Table 1, 16), which were most likely collected in the same period, ~1948–1949. Schaub Biol. 2 and Stuart B5W 3777 were later reclassified as *A. baumannii*, either based on DNA–DNA homology and sequencing of the *rpoB* gene and its flanking spacers [22,87], or by comparative analysis of the whole genome (GenBank: MTGG00000000.1) [102]. Schaub strain Eddy was also assigned to * A. baumannii* by PCR fingerprinting [103], although it is currently labelled *Acinetobacter* sp. pending further confirmation of its taxonomic status.

## ‘*Herellea*’, ‘*B. anitratum*’ and B5W were linked to each other

A link between members of the tribe ‘*Mimeae*’ described by De Bord and later adopted by Deacon and the novel species ‘*B. anitratum*’ proposed by Schaub and Hauber was first suggested by Ewing in 1949 (Fig. 1), who noted that the two cultures identified by Deacon as ‘*Herellea*’, Deacon 6-561 and Deacon 5-156A, were agglutinated by the ‘*B. anitratum*’ antiserum [104]. Both Deacon 6-561 and Deacon 5-156A, along with several other isolates, were once again reclassified as ‘*B. anitratum*’ based on their phenotypic features [85]. The authors argued that De Bord’s (1942) description of ‘*H. vaginicola*’ did not fit any of the ‘*B. anitratum*’ strains included in their study.

In 1950, Ferguson and Roberts underlined a connection between B5W and ‘*B. anitratum*’ (Fig. 1) [100]. They investigated 109 isolates that belonged to B5W, according to [97], or to ‘*B. anitratum*’, according to [92], including 18 isolates from Stuart’s collection, 6 isolates from Dr. Schaub’s collection and 85 new cultures. The isolates were classified into ten serotypes based on their capsular antigens [100]. In addition to Ferguson B5W 101 (corresponding to Schaub Biol. 1), 13 of Ferguson’s isolates have been preserved to date (Table 1, 17–19 and 99–108). Isolates B5W 3, B5W 71 (Stuart H1166) and B5W 72 (Stuart H606) were reclassified as *A. baumannii* based on the results of DNA–DNA homology and sequence analysis of the *rpoB* gene and its flanking spacers [22,43, 87]. Nonetheless, PCR fingerprinting with tRNA consensus primers characterized Ferguson’s isolate B5W 3 as * A. calcoaceticus* [103]. B5W 2, represented by ATCC 27224 and LMG 1059 [25,28], is also assigned as *A. baumannii* even though we could not find supporting documents. The remaining nine isolates, B5W 4, B5W 6, B5W 13, B5W 19, B5W 53, B5W 69 (Stuart H1168), B5W 79 (Stuart H1169), B5W 81 (Stuart H1167), and B5W 99 (Stuart W832), are currently designated as *Acinetobacter* sp., awaiting further classification.

## ‘*Moraxella lwoffi*’ and ‘*Moraxella glucidolytica*’: both were oxidase-negative and could be separated

In 1951, Piéchaud *et al*. proposed a new biovar under the species ‘*Moraxella lwoffi*’, named ‘var. *glucidolytica*’, to distinguish strains that were able to produce acid from glucose and other monosaccharides (Fig. 1) [105,106]. The authors also proposed that both B5W and ‘*B. anitratum*’ be transferred to the genus *Moraxella*. In line with this proposal, a very high similarity was noted between two isolates of B5W/‘*B. anitratum*’ and one isolate of ‘*Moraxella lwoffi* var. *glucidolytica*’ [107]. In 1952, Henriksen highlighted that the oxidase-positive variety of ‘*M. polymorpha*’, called ‘*M. polymorpha* var. *oxidans*’, appeared to be indistinguishable from ‘*Moraxella duplex* var. *nonliquefaciens*’ and should be included in the genus *Moraxella*. However, no isolates of this variety were later linked to *Acinetobacter*. Overall, Henriksen recommended that the relationship of other varieties of ‘*M. polymorpha*’, ‘*Herellea*’, ‘*B. anitratum*’ and B5W organisms to *Moraxella* should be decided when better evidence would be available [33]. In 1956, the ‘*glucidolytica*’ biovar was given species status as ‘*Moraxella glucidolytica*’ [108]. In addition, *M. lacunata*, ‘*M. duplex*’, ‘*Moraxella lwoffi*’ and ‘*M. glucidolytica*’ were each subdivided into two varieties, ‘var. *liquefaciens*’ and ‘var. *nonliquefaciens*’, based on their proteolytic ability (Table S3). Both Henriksen and Piéchaud *et al*. questioned the classification of ‘*Moraxella lwoffi*’ and ‘*M. glucidolytica*’ because both were oxidase negative (Fig. 1) [33,108].

## Piéchaud and Second’s isolates: from ‘*M. glucidolytica* var. *nonliquefaciens*’ to *A. baumannii* and *Acinetobacter pittii*; from ‘*Moraxella lwoffi* var. *nonliquefaciens*’ to *A. lwoffii*, *A. junii*, *Acinetobacter indicus* and *A. johnsonii*; from ‘*M. glucidolytica* var. *liquefaciens*’ to *Acinetobacter* genospecies 6 and *Acinetobacter haemolyticus*; and from ‘*Moraxella lwoffi* var. *liquefaciens*’ to *Acinetobacter higginsii* and *Acinetobacter haemolyticus*

Eighteen isolates that were included in Piéchaud and Second’s studies have been preserved until now. Piéchaud strain Rochefort (Table 1, 20) is now reclassified as *A. baumannii* (GenBank: CP000521.1 to CP000523.1) [8,43], Piéchaud strain Faucon (Table 1, 84) as *A. indicus* (GenBank: APRK00000000.1 and JACDQT000000000.1) [8,109], Piéchaud A165 (Table 1, 55) as *Acinetobacter* genospecies 6 (GenBank: APOK00000000.1) [8,43, 110], CIP 56.2 (Table 1, 85) as *A. higginsii* (GenBank: APPH00000000.1) [111] and Tanger H315 (Table 1, 62) as *A. johnsonii* [2,22, 43]. Piéchaud strains Gaillard, Valentin and de Croy (Table 1, 21, 24 and 25) were assigned to *A. baumannii* based on the sequences of their *rpoB* genes and flanking spacers [87].

According to the catalogue of micro-organims of the Biological Resource Center of Institut Pasteur, isolate CIP 52.90 (Table 1, 33) is now identified as *A. pittii*, CIP 53.66 (Table 1, 22) as *A. baumannii* and Piéchaud strains n° 1, 2, 3, 4, 7, 8 and 10 and isolate CIP 53.143 (Table 1, 36–43) as *Acinetobacter haemolyticus*, although the classification of the latter 10 isolates might need further confirmation [39]. Five more *Acinetobacter* isolates were deposited in the Collection de L’Institut Pasteur between 1951 and 1955 (Table 1). The identification of CIP 51.11 (Table 1, 72) as *A. lwoffii* was confirmed by genome sequencing (GenBank: APRU00000000.1) [38]. However, access to this strain has recently been paused at CIP [39], and only its subculture CNCTC 7857 (ANC 4568) is currently available [112]. CIP 51.14 and CIP 51.35 (Table 1, 51 and 52) are now described as *A. junii* while Haguenau 11.976 and Saint Quentin strain 2 (Table 1, 73 and 74) as *A. lwoffii* [39]. We could not find any articles or GenBank records supporting the identification of these four isolates.

Unlike most of the other *Acinetobacter* isolates cultured before 1955, the *A. indicus* Piéchaud strain Faucon exhibited minimum inhibitory concentrations of amoxicillin and ticarcillin at >256 mg l^−1^, ceftazidime at 2 mg l^−1^ and imipenem at 8 mg l^−1^ [109]. Sequence analysis revealed the presence of *bla*_OXA-58_, a carbapenemase gene, in the genome of CIP 53.82, where it was carried on a plasmid of 43,103 bp [109]. Piéchaud A165 was also found to be resistant to amikacin, kanamycin, netilmicin and tobramycin [113]. This was linked to the presence of a chromosomal gene, *aac(6′)-Ik*, a specific gene for *Acinetobacter* genospecies 6 [113,114].

## The switch towards ‘*Achromobacter lwoffi*’ and ‘*Achromobacter anitratum* (*anitratus*)’

In 1952, Brisou and Morichau-Beauchant proposed the transfer of ‘*Moraxella lwoffi*’, including var ‘*glucidolytica*’ (‘*B. anitratum*’), from the genus *Moraxella* into the genus *Achromobacter*, and the names ‘*Achromobacter lwoffi*’ and ‘*Achromobacter anitratum*’ were proposed (Fig. 1) [107]. The transfer of B5W, ‘*B. anitratum*’, ‘*Moraxella lwoffi*’ and ‘*M. glucidolytica*’ to the genus *Achromobacter* was again underlined by a few studies in 1953 (Fig. 1) [115,117]. Brisou also suggested that ‘*Neisseria winogradskyi*’ should be included in the genus *Achromobacter* [115,118]. The incorporation of several species of the genus *Alcaligenes* into the genus *Achromobacter* was recommended by Brisou but objected by H. Floch [115,117]. In line with Brisou’s recommendations, the switch from ‘*Alcaligenes metalcaligene*’ to ‘*Achromobacter metalcaligene*’ and from ‘*Alcaligenes marshallii*’ to ‘*Achromobacter marshallii*’ was later proposed (see below) [119,120]. It is worth mentioning that ‘*Achromobacter anitratum* (*anitratus*)’ was increasingly reported as the causative agent of a variety of human or animal infections [121,124]. According to Hugh and Reese, ‘*Achromobacter anitratus*’ and ‘*B. anitratum*’ were synonymous, with ‘*Achromobacter anitratus*’ being a legitimate published name [85].

## The genus *Acinetobacter*

In 1954, Brisou and Prévot coined the name *Acinetobacter* from the Greek word ‘ακινɛτοσ” (akinetos), meaning non-motile, to separate non-motile from motile microorganisms within the genus *Achromobacter* (Fig. 1) [16]. The proposed genus *Acinetobacter* was placed in the tribe ‘*Achromobactereae*’ and the family *Pseudomonadaceae*. Several species were proposed based on their phenotypic features, including ‘*Acinetobacter marshallii*’, ‘*Acinetobacter anitratum*’, ‘*Acinetobacter winogradskyi*’, *Acinetobacter lwoffi*, ‘*Acinetobacter viscosum*’ and ‘*Acinetobacter metalcaligenes*’ (Fig. 1) [16,119, 125]. While ‘*Acinetobacter marshallii*’ liquefied gelatine (which was linked to proteolytic activity) and reduced nitrates to nitrites, the other five species did not have the ability to liquefy gelatine, to reduce nitrates to nitrites or to produce indole or hydrogen sulphide (H2S). ‘*Acinetobacter anitratum*’ and ‘*Acinetobacter winogradskyi*’ were described as ‘carbohydrate lytic (glucidolytiques)’, while ‘*Acinetobacter marshallii*’, *Acinetobacter lwoffi*, ‘*Acinetobacter viscosum*’ and ‘*Acinetobacter metalcaligenes*’ were reported as ‘non carbohydrate lytic (non glucidolytiques)’.

In 1957, ‘*Acinetobacter anitratum*’ was designated as the type species of the genus *Acinetobacter* [44,125]. However, in 1958 and 1959, Klinge considered the genus *Acinetobacter* superfluous and rather placed ‘*Moraxella lwoffi*’ in the genus *Alcaligenes* and ‘*M. glucidolytica*’ in the genus *Achromobacter* [126,127]. Courtieu *et al*. also argued that it was premature to draw a definitive conclusion about the classification of ‘*Moraxella Iwoffi*’ and ‘*M. glucidolytica*’ [128]. On the other hand, the separation of ‘*Moraxella lwoffi*’ and ‘*M. glucidolytica*’, which were oxidase-negative and penicillin-resistant, from other oxidase-positive and penicillin-sensitive *Moraxella* species and their classification as *Acinetobacter lwoffi* and ‘*Acinetobacter anitratum* (*anitratus*)’, respectively, was again recommended by Henriksen in 1960 [129]. This classification was also used by Steel and Cowan in 1964 who proposed that ‘*Diplococcus mucosus*’, ‘*B. anitratum*’, ‘*Moraxella lwoffi* subsp. *glucidolytica*’ and ‘*H. vaginicola*’ could be grouped under ‘*Acinetobacter anitratus*’ (type strain: NCTC 7844, corresponding to Schaub Biol. 1) and that ‘*Moraxella lwoffi*’ could be reclassified as *A. lwoffii* (type strain: NCTC 5866, corresponding to Lwoff 1) [37]. The authors also proposed that ‘*Bacillus mallei*’ and ‘*Haemophilus parapertussis*’ be renamed ‘*Acinetobacter mallei*’ (type strain: NCTC 10230) and ‘*Acinetobacter parapertussis*’ (type strain: NCTC 5952), respectively. However, it is now known that the latter two isolates actually belong to *Burkholderia mallei* and *Bordetella parapertussis*, respectively [130]. The use of ‘*A. lwoffii*’ and ‘*A. anitratus*’ was then followed by a few other research groups (e.g. [131,133]).

In 1967, Thornley proposed a revised definition of the genus *Acinetobacter* as follows: Gram-negative or Gram-variable, non-motile, coccoid rods, short rods or cocci, often in pairs; able to grow aerobically on ordinary nutrient media without the addition of serum; colonies whitish or creamy, producing no fluorescent pigment; catalase-positive; sugars oxidized or not attacked at all; and arginine test negative (Fig. 1) [93]. In 1968, Baumann *et al*. published a comprehensive study on the nutritional and biochemical properties of 106 oxidase-negative *Moraxella* isolates, also called the ‘*Mima-Herellea-Acinetobacter*’ group (Fig. 1). Based on the results of 56 tests, a modified description of the genus *Acinetobacter* [16] was presented, and a proposal was made to place all the oxidase-negative *Moraxellas* (‘*M. calco-aceticus*’, ‘*Alcaligenes hemolysans*’, ‘*M. polymorpha*’, ‘*Moraxella lwoffi*’, ‘*H. vaginicola*’ and ‘*B. anitratum*’) in this genus [2]. Since the epithet ‘*calcoaceticus*’ was the first published name for an organism of this genus, Baumann *et al*. proposed using the name ‘*A. calcoaceticus*’ instead of ‘*Acinetobacter anitratum*’ to refer to the type species of this genus. Two other species, *Acinetobacter lwoffi* (Audureau) and ‘*Acinetobacter hemolysans*’ (Henriksen), and one subspecies, ‘*Acinetobacter hemolysans* subsp. *haemolyticus*’ (Stenzel and Mannheim), were provisionally recognized by this study [2].

The genus *Acinetobacter* was officially acknowledged by the International Association of Microbiological Societies in 1970, as stated in the Minutes of the Meeting of a subcommittee on nomenclature of *Moraxella* and allied bacteria (Fig. 1) [134]. The subcommittee also agreed that *Neisseria*, *Moraxella* and *Branhamella* could be tentatively united in the family *Neisseriaceae* and that the genus *Acinetobacter* could be temporarily associated with this family. In 1974, the genus *Acinetobacter* was listed in the eighth edition of Bergey’s Manual of Determinative Bacteriology, with only one formally described species, *A. calcoaceticus* [135]. Two *Acinetobacter* species were listed in the 1980 ‘Approved List of Bacterial Names’ (Fig. 1) [17], with *A. calcoaceticus* representing strains that produced acid from sugars and *A. lwoffii* for those that did not (Fig. 1) [5].

## From ‘*Diplococcus mucosus*’ to *A. baumannii*

The name ‘*D. mucosus*’ was proposed by Von Lingelsheim in 1906 to distinguish Gram-negative diplococci and tetrads with capsules that grew on ordinary agar and gelatin as fairly large (3–4 mm wide), round and slightly confining colonies (Fig. 1) [136]. In 1953, Seeliger demonstrated a notable similarity between several ‘*D. mucosus*’ isolates and a set of reference strains of ‘*B. anitratum*’ and proposed that the two names were synonyms (Fig. 1) [137]. Although this proposal was rejected by a few other studies [93,121], the name ‘*Achromobacter mucosus*’ (as a derivative from ‘*D. mucosus*’) was once again proposed in 1962 to describe organisms belonging to ‘*B. anitratum*’, ‘*H. vaginicola*’ or ‘*Moraxella lwoffi* var. *glucidolytica*’ (see below) [138]. On the contrary, Hugh and Reese stated that ‘*Achromobacter mucosus*’ should actually be re-identified as ‘*B. anitratum*’ [85]. Likewise, Baumann *et al*. declined the name ‘*Achromobacter mucosus*’ (Mannheim and Stenzel) and regarded it as a synonym of *A. calcoaceticus* (‘*M. calco-aceticus*’) [2]. The ‘*D. mucosus*’ strains studied by Veron *et al*. were able to grow at a temperature of 42 °C although this was mainly related to a selected bacterial subpopulation [139]. Similarly, the isolates studied by Klinge, and the one described by Mannheim and Stenzel as ‘*Achromobacter mucosus*’ also multiplied at 44 °C [127,136].

One of Seeliger’s ‘*D. mucosus*’ isolates, D5/62 (Table 1, 23), is now reclassified as *A. baumannii* based on DNA–DNA hybridization and the *rpoB* gene sequence analysis [22,87]. The genome of ATCC 17957, a subculture of D5/62, is now available at the ATCC (https://www.atcc.org/products/17957). The LMG 1014 culture contained two unstable colony types [23]. V 2240-52 (Table 1, 111) is another isolate deposited by Seeliger as ‘*D. mucosus*’, which is currently designated as *Acinetobacter* sp. until further classification [28,85].

We found seven other isolates in the NCTC [130] that were collected before 1948 and were originally deposited as ‘*D. mucosus*’ but are currently classified as *A. baumannii* (Table 1, 9–11 and 92–95). These isolates were also described as ‘*Achromobacter* (*Bacterium*) *anitratum*’, ‘*A. calcoaceticus* var. *anitratus*’ or ‘*A. anitratus*’ [40,140,142]. The classification of three of them, i.e. NCTC 7364 (strain Carr), NCTC 7412 (strain Jones) and NCTC 7422 (strain Ayers), as *A. baumannii* was confirmed by genome sequencing (GenBank: LT605059.1 and LT605060.1 for NCTC 7364, UFRP00000000.1 for NCTC 7412 and AIED00000000.1 for NCTC 7422) [143]. The methyl red test was reported to be positive for NCTC 7422, contradicting the result of the other isolates [141]. A positive methyl red test, i.e. a decrease in pH below 4.4, is very unlikely for *Acinetobacter* [132]. We found no additional information supporting the classification of the other four isolates, namely NCTC 7250 (strain Bellairs), NCTC 7363 (strain Poultney), NCTC 7461 (strain Crook) and NCTC 7462 (strain Hourigan), as *Acinetobacter* sp.

## NCTC 9427: from ‘*Achromobacter* (*Bacterium*) *anitratum*’ to *Acinetobacter haemolyticus*

We found another isolate, NCTC 9427(82 Groombridge), which was first reported as ‘*Achromobacter* (*Bacterium*) *anitratum*’, ‘*A. calcoaceticus* var. *anitratus*’ or ‘*A. anitratus*’ (Table 1, 112) [40,141, 142] but is currently preserved as *Acinetobacter haemolyticus* [130]. NCTC 9427 was catalase-negative, differentiating it from the catalase-positive *Acinetobacter* isolates [141]. We could not find any taxonomic study to support the classification of this isolate as *Acinetobacter haemolyticus*.

## Billing’s soap-tolerant isolates: from ‘*Achromobacter anitratum* var. *saponiphilum*’ to *Acinetobacter haemolyticus*

A soap-tolerant organism was reported in 1955 as a new variety of ‘*B. anitratum*’ [144]. An antigenic relationship was detected between the soap-tolerant isolates, provisionally named ‘*Achromobacter anitratum* var. *saponiphilum*’, and a number of reference ‘*B. anitratum*’ isolates. One of the soap-tolerant isolates, Billing A3;ST3 (Table 1, 44), was reclassified as *Acinetobacter haemolyticus* based on DNA–DNA homology [22,43]. Another isolate, Billing A1;ST1 (Table 1, 110), is currently maintained as *Acinetobacter* sp. or *Acinetobacter haemolyticus* in a few national culture collections. NCTC 7976 (Table 1, 71) was included in Billing’s study as a reference strain for ‘*Moraxella lwoffi* var. brevis’. NCTC 7976 is currently recognized as *A. lwoffii*, although it has only been characterized phenotypically (see below) [93]. We found another ‘*B. anitratum*’ isolate deposited by Billing, B4 (Table 1, 113), which was most likely also part of this study. This isolate is now classified as *Acinetobacter* sp. until further considerations.

## Soil isolates reclassified from ‘*N. winogradskyi*’ and ‘*Micrococcus cerificans*’ to *A. calcoaceticus* and from *A. calcoaceticus* to *Acinetobacter baylyi*

Eighteen isolates, represented by strain CIP 66.32 (Table 1, 4), were cultured from soil and described by Lemoigne *et al*. in 1952 under the name ‘*N. winogradskyi*’, although the authors also considered classifying them as ‘*Moraxella lwoffi*’ in line with Piéchaud’s recommendations [118]. CIP 66.32 is currently known to be *A. calcoaceticus* (GenBank: AMIW00000000.1) [22,101]. Another isolate, H.O.1 (Table 1, 5), was cultured from hexadecane-mineral salts enrichments in 1959 [145]. This isolate was described as an alkane-oxidizing Gram-negative micro-coccus and was first named ‘*Micrococcus cerificans*’ [145,146]. H.O.1 was later considered as a typical strain of ‘*B. anitratum*’ [85] and finally identified as *A. calcoaceticus* (GenBank: AMIY00000000.1) [22,101].

In 1961, Taylor and Juni described a soil bacterium, strain BD-4 (Table 1, 87), able to grow well on a mineral salt medium with a single carbon source [147]. BD-4 was found to be similar to the ‘*N. winogradskyi*’ bacterium reported by Lemoigne in 1952 [118]. BD-4 was later classified as *A. calcoaceticus* (‘*B. anitratum*’) in 1969 [148] and finally identified as *A. baylyi* (GenBank: AMIC00000000.1) [101]. A mutant of BD-4, first designated BD413 and later named ADP1 (Table 1, 88), was obtained by ultraviolet irradiation and has been widely used as a model organism for natural transformation, genetic analysis and genome engineering (GenBank: CR543861.1) [148,151].

## Courtieu’s isolates: from ‘*Herellea saponiphilum*’ and ‘*Herellea caseolytica*’ to *Acinetobacter haemolyticus*, from ‘*Herellea lwoffi*’ to *A. junii* and from ‘*H. vaginicola*’ to *A. baumannii* and *A. pittii*

We found five isolates collected by Courtieu between 1955 and 1957 according to our interpretation of the names of the isolates (Table 1, 26, 34, 45, 46 and 53). Courtieu 55.071.323 was reclassified from ‘*Herellea saponiphilum*’ to ‘*M. glucidolytica* var. *liquefaciens*’ to *Acinetobacter haemolyticus* (GenBank: ADMT00000000.1), Courtieu 56.073.110 from ‘*Herellea lwoffi*’ to ‘*Moraxella lwoffi* var. *nonliquefaciens*’ to *A. junii* [28], Courtieu 57.071.066 from ‘*H. vaginicola*’ to *A. baumannii* [28], Courtieu 57.071.228 from ‘*H. vaginicola*’ to *A. pittii* (GenBank: APQP00000000.1) and Courtieu 57.073.192 from ‘*Herellea caseolytica*’ to *Acinetobacter haemolyticus* (GenBank: AMJB00000000.1).

## Reclassification of NCDO 1092, obtained from an animal sample, from ‘*Alcaligenes haemolysans*’ to *Acinetobacter* sp.

In 1957, a Gram-negative cocco-bacillus, NCDO 1092 (Table 1, 115), was isolated from cow’s teat [75]. NCDO 1092 was found to be closely related to strain NCTC 5043, a reference strain for ‘*Alcaligenes haemolysans*’ (see above), although it did not liquefy gelatin while NCTC 5043 did [75]. The three ‘*Acinetobacter haemolyticus*’ isolates described by Piéchaud (strains 3, 7 and 10) were also able to liquefy gelatin [108]. NCDO 1092, first labelled as ‘*Alcaligenes haemolysans*’, is currently deposited as *A. calcoaceticus* [24], although this classification is probably incorrect, and further taxonomic analysis is needed.

## Brisou’s isolates: from ‘*N. winogradskyi*’ to *A. pittii*, from ‘*Alcaligenes metalcaligenes*’ to *A. johnsonii*, from ‘*Achromobacter anitrata*’ to *Acinetobacter bereziniae* and from ‘*Achromobacter venosum*’ to *A. lwoffii*

In addition to Brisou 65 that was mentioned above, we found five isolates that were isolated in or before 1957 and deposited by Brisou in the NCIB. Brisou 12 (Table 1, 35) was described as ‘*N. winogradskyi*’, ‘*Achromobacter winogradskyi*’ or ‘*Acinetobacter winogradskyi*’ before it was finally assigned to *A. pittii* [22,42, 43, 87]. Brisou 63 (Table 1, 59) was first classified as ‘*Alcaligenes metalcaligenes*’ but is currently identified as *A. johnsonii* [22,42, 43]. The classification of Brisou 64 (Table 1, 80) was changed from ‘*Achromobacter anitratum*’ to *A. bereziniae* (GenBank: APQG00000000.1) [53]. Brisou 66 (Table 1, 114) was first named ‘*Alcaligenes metalcaligenes*’ or ‘*Acinetobacter metalcaligenes*’ while it is now deposited as *Acinetobacter* sp., pending further classification. Brisou 78 (Table 1, 75) was first described as ‘*Achromobacter venosum*’ and later changed to *A. lwoffii* (confirmed by *rpoB* sequencing according to the Culture Collection University of Gothenburg). The motility of Brisou 78 should be verified since ‘*Achromobacter venosum*’ was described as motile [61].

## Cary’s study: from ‘*M. polymorpha*’ and ‘*Herellea* sp.’ to *Acinetobacter* sp.

In 1958, a slide agglutination technique was proposed for rapid differentiation of ‘*M. polymorpha*’ and ‘*Herellea*’ from the *Neisseriae* [152]. Five of the isolates used in this study (two ‘*M. polymorpha*’ and three ‘*Herellea*’ sp.) are currently available at the ATCC as *Acinetobacter* sp. (Table 1, 27 and 116–119). The two ‘*M. polymorpha*’ isolates, 24 (ATCC 142912) and 44 (ATCC 14291), belonged to subgroup B2 according to the numerical phenotypic analysis of Baumann *et al*., while the ‘*Herellea*’ isolate CDC 1847 (ATCC 14290) was part of subgroup A1 [2]. Based on whole-genome sequencing, isolate 499 (ATCC 14293), one of the ‘*Herellea*’ sp. isolates, should be assigned as *A. baumannii* [28]. On the other hand, no taxonomic information was found on the last ‘*Herellea*’ isolate, CDC 1856 (ATCC 14294). Although it seems likely, the ATCC records of isolates 24, 44 and 499 did not state if they corresponded to CDC 24, CDC 44 and CDC 499, respectively.

## Kambou and Enjalbert’s isolates: from ‘*Achromobacter marshallii*’ to *A. baumannii* and provisionally from ‘*Achromobacter delictatulum*’ to *A. johnsonii*

Seven isolates, 4.589, 88.860, 87.019, 87.059, 88.080, 88.125 and 88.504 (Table 1, 28, 60 and 120–124), were deposited in the NCIB database by Enjalbert with reference to Kambou thesis, Toulouse University [24,153]. Kambou 88.504 is now reclassified as *A. baumannii* based on whole-genome sequencing (GenBank: JAMOLC000000000.1) [96] while Kambou 87.019 is currently considered to be *A. johnsonii* although we could not find a confirming taxonomic study or GenBank record. LMG 1066, a subculture of Kambou 88.080, has two stable colony types [25].

## Other short-lived names

In 1961, Lautrop proposed transferring ‘*B. anitratum*’ to the genus *Cytophaga* under the name ‘*Cytophaga anitrata*’ (Schaub and Hauber) comb. nov.’ [154]. This suggestion was later rejected by Seeliger *et al*., who advised transferring it to the genus ‘*Lingelsheimia*’ gen. nov [155]. In 1964, Lwoff used the name ‘*Moraxella anitrata*’ [156]. In the same year, Mitchell and Burrell proposed that ‘*Mima anitratum*’ should include ‘*H. vaginicola*’, ‘*B. anitratum*’ and other related organisms that ferment certain carbohydrates, while ‘*Mima lwoffi*’ should cover organisms that do not ferment carbohydrates including ‘*Moraxella lwoffi*’ and ‘*M. polymorpha*’ [157]. However, all these names were overlooked by most of the subsequent studies.

## Lautrop isolates

Lautrop A3 was an isolate that could be traced back to a study performed in 1953 (Table 1, 58) [158]. The isolate was first described as ‘*Cytophaga lwoffi*’ and later placed in the genus *Acinetobacter* [2,43]. Lautrop A3 was then assigned into genospecies 7 (*A. johnsonii*) based on DNA–DNA homology [22]. Three other isolates were deposited by Lautrop in the NCTC database in 1961 or 1962 (Table 1, 29, 133 and 134). They were all described as *A. baumannii* although their identification could not be confirmed, especially since the biochemical features of two isolates, Lautrop A-4 (represented by NCTC 10290) and Lautrop B5W (NCTC 10292), were different from those of the third one, Lautrop A-15 (NCTC 10291) [40].

## ‘*B. anitratum*’ in animal samples

The occurrence of ‘*B. anitratum*’ in animals was first reported in 1950, where the features of two isolates obtained from foal carcasses were described [159]. However, these isolates have most likely been lost and we could not find any studies verifying their identity. In 1961, Ellis published a study on the recovery of ‘*B. anitratum*’ (‘B5W’) isolates from animal samples [160]. Two of Ellis’ isolates are preserved to date (Table 1, 126 and 127), both labelled as *Acinetobacter* sp. although limited taxonomic analysis was performed [85,161]. Ellis isolates 1972 and 2409 were recovered from chicken and bovine, respectively.

## Stenzel and Mannheim isolates: from ‘*Achromobacter mucosus*’ to *A. baumannii*, from ‘*Achromobacter conjunctivae*’ to *Acinetobacter colistiniresistens*, from ‘*Achromobacter haemolyticus*’ to *Acinetobacter haemolyticus*, from ‘*Achromobacter metalcaligenes*’ to *A. johnsonii* and *A. lwoffii* and from ‘*Achromobacter citroalcaligenes*’ to *A. junii*

In 1962 and 1963, Stenzel and Mannheim described five species and two subspecies in the genus *Achromobacter* [120,138]. The first species, ‘*Achromobacter mucosus*’, corresponded to ‘*B. anitratum*’, ‘*H. vaginicola*’ and ‘*Moraxella lwoffi* var. *glucidolytica*’. The authors considered the epithet ‘*mucosus*’, sub-cultured from ‘*D. mucosus*’ [136], to be the earliest valid description of this group of glucidolytic and non-proteolytic microorganisms. Stenzel and Mannheim strain 3516/60 (Table 1, 30) was selected as the neotype strain of ‘*Achromobacter mucosus*’. However, the neotype strain of ‘*Achromobacter mucosus*’ and the type strains of ‘*Achromobacter conjunctivae*’ and ‘*Achromobacter haemolyticus* subsp. *haemolyticus*’ (see below) were all re-identified as ‘*B. anitratum*’ [85]. The whole-genome sequencing of CCUG 890, a subculture of Stenzel and Mannheim strain 3516/60, enabled us to reclassify it as *A. baumannii* (GenBank: JAMOLB000000000.1) [96].

The species ‘*Achromobacter conjunctivae*’ was described as a specific causative agent of subacute conjunctivitis in man. It was not pathogenic for mice, produced haemolysis on blood agar, did not grow in an ammonium-ethanol medium or on Leifson’s agar and possessed a specific major K-antigen that could be used for serological identification. The type strain, Stenzel and Mannheim strain P544/60 (Table 1, 83), was later assigned to *A. colistiniresistens* (GenBank: APRT00000000.1) [162].

Stenzel and Mannheim strains 2446/60 and 2181/60 (Table 1, 47 and 48) were selected as the type strains of ‘*Achromobacter haemolyticus* subsp. *haemolyticus*’ (also named ‘*Achromobacter haemolyticus* subsp. g*lucidolytica*’) and ‘*Achromobacter haemolyticus* subsp. *alcaligenes*’, respectively. The ‘*Achromobacter haemolyticus*’ isolates resembled ‘*Achromobacter conjunctivae*’ in their haemolytic activity and inability to grow at 45 °C, but they were able to grow on Leifson’s agar and in an ammonium-ethanol medium. These two strains were later reclassified as *Acinetobacter haemolyticus* (GenBank: UFRR00000000.1 and UFRT00000000.1) [8,101, 110, 163]. The proposed neotype strain of ‘*Achromobacter metalcaligenes*’, corresponding to ‘*Moraxella lwoffi*’, ‘*M. polymorpha*’ and ‘*Alcaligenes metalcaligenes*’, was Stenzel and Mannheim strain 3865/60 (Table 1, 61), which is now the type strain of *A. johnsonii* (GenBank: APON00000000.1 and AMJD00000000.1) [8,101, 110].

Stenzel and Mannheim strain P790/60 (Table 1, 76) was described as a variety of ‘*Achromobacter metalcaligenes*’ that was able to attack glucose, galactose, arabinose and xylose after a marked delay. This isolate is now a member of *A. lwoffii* (GenBank: AMJG00000000.1 and APRY00000000.1) [8,101, 110]. ‘*Achromobacter citroalcaligenes*’ was distinguished from ‘*Achromobacter metalcaligenes*’ by its ability to attack ammonium citrate promptly with strong alkalinity production. Stenzel and Mannheim strain 2723/59 (Table 1, 54) is now the type strain of *A. junii* (GenBank: APPX00000000.1 and AMJF00000000.1) [8,101, 110].

## More isolates reclassified from ‘*H. vaginicola*’ or ‘*Herellea*’ sp. to *A. baumannii*, *Acinetobacter haemolyticus* or *Acinetobacter* sp.

Several ‘*Herellea*’ isolates, collected between 1952 and 1966, are now recognized as *Acinetobacter* spp., including Kass 13860 (ATCC 17961), Kass 61-29261 (ATCC 27231), Kass 1170 (ATCC 27233), Kass 50372 (ATCC 27235), Kass 54651 (ATCC 27236), 4935 (ATCC 27234), 4936 (ATCC 27232), 1043 (ATCC 27237), 7186 (ATCC 27238), 8886 N (ATCC 27239), 9358 (ATCC 27240), 8647 (ATCC 27241), 9391 (ATCC 27242), 9378 (ATCC 27243), 9458 (ATCC 27244), 9346 (ATCC 27245), 9469 N (ATCC 27246), 9092 N (ATCC 27247), 8870 N (ATCC 27248), 47-8628 (ATCC 27249), 47-8643 (ATCC 27250), 47-8928 (ATCC 27251), 47-8922 (ATCC 27252), 47-9192 (ATCC 27253), 47-9253 (ATCC 27254), King 6801 (ATCC 19684), King 7411 (ATCC 19683) and ATCC 11959 (Table 1, 31, 49, 109, 128–132, 135–144, and 146–155). The isolates of E. H. Kass were numbered according to a system used by the Boston City Hospital while the remaining isolates, submitted to ATCC by W. H. Ewing, E. O. King or W. B. Cherry, could probably be correlated to the CDC numbering system [164].

Kass 13860 (Table 1, 31), also known as CDC 7788, was reclassified as *A. baumannii* based on whole-genome sequencing (GenBank: CP065432.1-CP065434.1) [22,157, 165]. The *sul2* gene was detected on the chromosome of ATCC 17961, a subculture from Kass 13860 [96]. Strain 9458 (ATCC 27244) was reclassified from ‘*H. vaginicola*’ to *Acinetobacter haemolyticus*, also based on whole-genome sequencing (Table 1, 49, GenBank: ABYN01000000.1 and AMJC00000000.1) [101,163, 164]. The remaining isolates are assigned as *Acinetobacter* sp. until further classification. Many of these isolates were studied by Marcus *et al*. where they were found to be oxidase negative, non-motile, nitrate reduction negative and mostly citrate utilization positive [164].

Isolates Kass 61-29261, Kass 1170, Kass 50372 and Kass 54651 (Table 1, 129–132) were among a total of 11 clinical isolates collected from 9 bacteraemia patients in the USA with a mortality rate of 70% [166]. According to the literature, King 6801 and King 7411 (Table 1, 146 and 147) were able to grow at 42 °C and both demonstrated motility in Cystine Trypticase semisolid medium [167]. However, Hugh and Reese could not confirm motility or detect flagella on cells of these two strains [85]. ATCC 11959 (Table 1, 148) was one of the earliest strains used to isolate and describe the characteristics of *Acinetobacter*-specific phages (Fig. 1) [168].

## Additional strains from the 1960s

We found nine isolates deposited by or correlated to S. D. Henriksen (4233/62, NCDC KC764 and Lausanne 397), R. W. Kinney (14A2), N. Walker (NCIMB 9689), J. E. Johnson (CDC 7827), U.S. Food and Drug Administration (FDA PCI-3), Boston City Hospital (FDA PCI 1788) and Y. Nakasuji (A43), which were most likely collected in the 1960s and are now classified as acinetobacters. Henriksen strain 4233/62 (Table 1, 86), first named as ‘*Alcaligenes haemolysans*’, ‘*Achromobacter haemolyticus*’ and ‘*Moraxella lwoffi* var. *liquefaciens*’, is now recognized as the type strain of *A. higginsii*, corresponding to *Acinetobacter* genomic species 16 (GenBank: AMJM00000000.1 and APRN00000000.1) [111,169]. The intrinsic OXA gene in the genome of strains CIP 56.2 (see above) and 4233/62, designated *bla*_OXA-287_ (NCBI Reference Sequence: NG_050609.1) and *bla*_OXA-288_ (NG_049585.1), respectively, shared less than 90% identity with each other (unpublished data), which might reflect a relatively low intraspecies genomic nucleotide identity for the species *A. higginsii* [111]. NCDC KC764 and Lausanne 397 (Table 1, 78 and 79) were reclassified from ‘*Moraxella lwoffi*’ to *A. lwoffii* based on genome sequencing of ATCC 17985, a subculture of NCDC KC764 [28], and by DNA–DNA homology, *gyrB* and *rpoD* sequence analyses for subcultures of strain Lausanne 397 [22,170].

Kinney 14A2 (Table 1, 125) was isolated from soil with 2-leucine as the sole carbon and nitrogen source and was first recognized as the type strain of a novel species named ‘*Brevibacterium leucinophagum*’ [171]. However, the morphological, physiological and electron microscopy features of ATCC 13809, a subculture of Kinney 14A2, indicated that it was Gram-negative and should probably be placed in the genus *Acinetobacter* [172]. The authors reported a close relationship of this strain to *Acinetobacter* phenon 2 of Thornley (see below). Although ATCC 13809 was later reported as *A. baumannii* [173], species identification of this strain requires further validation.

In 1964, Claus and Walker cultured a soil bacterium, corresponding to NCIMB 9689 (Table 1, 145), that was able to grow with toluene as the sole carbon source [174]. A subculture of this isolate was later subjected to genome sequencing and our preliminary analysis of the results indicated that it might represent a new species (unpublished data). Strain CDC 7827 (Table 1, 32) was reclassified from ‘*M. polymorpha*’ to *A. baumannii* based on DNA–DNA homology and *rpoB* sequence analyses [22,87, 157]. FDA PCI-3 and FDA PCI 1788 (Table 1, 159 and 160) were reclassified from ‘*Bodenheimer’s bacillus*’ to *Alcaligenes* sp., then to ‘*B. anitratum*’ and finally to *Acinetobacter* sp. [28,85, 94] and from ‘*Herellea* sp.’ to *A. baumannii*, respectively [28,85]. We could not find other studies supporting the classification of these two strains. Nakasuji A43 (Table 1, 161) is now deposited as * A. calcoaceticus* [175]. According to this catalogue, the methyl red test for IFO 13006, a subculture of Nakasuji A43, was positive, as it was for IFO 12552 (representing the type strain of *A. calcoaceticus*). However, our preliminary analysis of the publicly available whole genome of IFO 13006 demonstrated a close relationship to *Acinetobacter vivianii* (unpublished data; GenBank: BAABSW000000000.1) [176].

## Thornley study: from ‘*Achromobacter mucosus*’ to *A. baumannii*, from ‘*Achromobacter conjunctivae*’ to *A. colistiniresistens*, from ‘*Achromobacter haemolyticus*’ to *Acinetobacter haemolyticus*, from ‘*Achromobacter metalcaligenes*’ to *A. johnsonii* and *A. lwoffii* and from ‘*Achromobacter citroalcaligenes*’ to *A. junii*

A numerical taxonomic study of the genus *Acinetobacter* was performed in 1967 based on the morphological and biochemical properties of 77 named strains that were obtained from national or private collections and were mostly from genera related to *Achromobacter* and 120 isolates of Gram-negative or Gram-variable non-motile coccoid rods cultured by the author from poultry carcasses [93]. The author’s isolates, formerly assigned as *Achromobacter*, were designated MJT followed by the experiment and strain numbers. After the calculation of the matrix of similarity coefficients between all pairs of strains, the strains were sorted into clusters, called phenons.

Isolates of phenons 2, 3 and 4 were classified as *Acinetobacter*, with phenons 2 and 3 oxidizing pentoses and hexoses while phenon 4 did not. Overall, phenon 2 included isolates that were formerly described as ‘*Achromobacter lacticum*’, ‘*B. anitratum*’, ‘*Herellea*’ sp., ‘*D. mucosus*’, a glucose-oxidizing form of ‘*Moraxella lwoffi*’, ‘*Achromobacter anitratus*’ or ‘*A. anitratum* (*anitratus*)’. *A. baumannii* 4H and 6H (see above) [78] were members of phenon 2. Schaub isolates 90 (*Acinetobacter* sp.), 93 (*A. baumannii*), biol.2 (*A. baumannii*) and Eddy (*Acinetobacter* sp.); Ferguson isolates B5W 3 (*A. baumannii*), B5W 72 (*A. baumannii*) and B5W 99 (*Acinetobacter* sp.); and Stuart A267 (*A. nosocomialis*) were also assigned into phenon 2. Brisou 64, which was later reclassified as *A. bereziniae* (see above), also belonged to phenon 2 [22,53]. In addition, one of the phenon 2 ‘*D. mucosus*’ isolates, Klinge E2241/60 (Table 1, 156), has been preserved to date. Klinge E2241/60 is currently designated as *Acinetobacter* sp. pending further taxonomic analysis.

Only one of the phenon 3 isolates, MJT/F5/158 (Table 1, 158), is available today [177]. It is preserved as *A. baumannii* although we could not find any information to support this. In fact, the overall classification of phenon 3, including this isolate, as *Acinetobacter* should be reconsidered since 96% of the phenon 3 isolates were oxidase-positive. The isolates in phenon 4 were formerly described as ‘*Mima*’ sp., ‘*Moraxella lwoffi*’ (not producing acid from sugars), ‘*Achromobacter venenosum*’ and ‘*Alcaligenes viscosus* (*viscolactis*)’. Some of the phenon 4 isolates were oxidase-positive and therefore not *Acinetobacter*. Nonetheless, phenon 4 included NCTC 5866=Lwoff 1 (*A. lwoffii*), NCTC 5867=Lwoff 2 (*A. lwoffii*), NCTC 7976 (*A. lwoffii*), NCIB 9022=Brisou 78 (*A. lwoffii*), NCIB 8596=DB264 (*Acinetobacter* sp.) and NCIB 8154=ATCC 9036 (*A. johnsonii*). This phenon also included Klinge 950/56 and MJT/FS/122 (Table 1, 77 and 157), which are now classified as *A. lwoffii* and *Acinetobacter* sp., respectively.

## The earliest recognition of *Acinetobacter* as a significant opportunistic pathogen

The pathogenic capability of *Acinetobacter* was first noted in the late 1930s, in the form of *A. lwoffii* and *A. johnsonii* conjunctivitis [36]. Other species, such as *A. baumannii*, *A. junii*, *A. indicus*, *A. pittii*, *A. haemolyticus*, *A. higginsii* and *A. bereziniae*, were detected in a variety of clinical samples, mainly urine and cerebrospinal fluid but also respiratory secretions, blood, faeces, pus and burn swabs, as early as the 1940s and 1950s [92,100, 108]. Many of these isolates were hospital-related and although the infections were sometimes severe and even fatal, *Acinetobacter* was generally regarded as a harmless commensal bacterium [178]. In the 1960s, *Acinetobacter* started to establish itself as an emerging opportunistic nosocomial pathogen [179,181]. Contamination of hospital respirators and being already debilitated by other medical conditions were suggested as potential risk factors [182,183]. Until the early 1970s, nosocomial *Acinetobacter* infections were often treated successfully with gentamicin, minocycline, nalidixic acid, ampicillin or carbenicillin, either as single agents or in combinations [4]. Overall, the temporal evolution of *Acinetobacter* has revealed a concerning pattern of several species emerging as potential opportunistic pathogens, among which *A. baumannii* is by far ahead in antibiotic resistance [184,185].

## Conclusion

We identified several species names that were used to describe organisms of the genus *Acinetobacter* but lost their standing in the official bacterial nomenclature on 1 January 1980, including ‘*M. calco-aceticus*’, ‘*D. mucosus*’, ‘*Achromobacter mucosus*’, ‘*B. anitratum*’, ‘B5W’, ‘*H. vaginicola*’, ‘*M. glucidolytica*’, ‘*N. winogradskyi*’, ‘*Achromobacter winogradskyi*’, ‘*Acinetobacter winogradskyi*’, ‘*Achromobacter marshallii*’, ‘*Acinetobacter marshallii*’, ‘*Achromobacter anitratus*’, ‘*Acinetobacter anitratum*’, ‘*Achromobacter anitratum* var. *saponiphilum*’, ‘*Alcaligenes viscosus*’, ‘*M. polymorpha*’, non-proteolytic ‘*Moraxella lwoffi*’, proteolytic ‘*Moraxella lwoffi*’, ‘*M. glucidolytica*’ (proteolytic), ‘*Achromobacter lwoffi*’, ‘*Achromobacter metalcaligenes*’, ‘*Achromobacter haemolyticus*’ (including subsp. *haemolyticus* and subsp. *alcaligenes*), ‘*Achromobacter citroalcaligenes*’, ‘*Achromobacter conjunctivae*’ and ‘*M. cerificans*’.

To our knowledge, *A. calcoaceticus* Beijerinck 1 (isolated ≤1911) is the oldest available strain of the genus *Acinetobacter*. ATCC 9036 (≤1936), Audureau strain bacteroides (≤1940) and possibly DB264 (1931) are the oldest maintained strains for *A. johnsonii*; Lowff 1 (≤1939), Lwoff 2 (≤1939) and Audureau strain brevis (≤1940) for *A. lwoffii*; Allen 4H (≤1944), Allen 6H (≤1944) and Deacon 6-561 (≤1945) for *A. baumannii*; Rubinsten strain 1(1947) for *A. junii*; Stuart A267 (≤1949) for *A. nosocomialis*; NCIB 8250 (≤1951) for *A. guillouiae*; Piéchaud strain Faucon (≤1951) for *A. indicus*; CIP 52.90 (1952) for *A. pittii*; Piéchaud A165 (≤1952) for *Acinetobacter* genospecies 6; Piéchaud strains n° 1, 2, 3, 4, 7, 8 and 10 (≤1953) and CIP 53.143 (≤1953) for *Acinetobacter haemolyticus*; CIP 56.2 (1955) for *A. higginsii*; Brisou 64 (≤1957) for *A. bereziniae*; Brisou 65 (≤1957) for *A. pseudolwoffii*; BD-4 (1961) for *A. baylyi*; and Stenzel and Mannheim P544/60 (≤1962) for *A. colistiniresistens*.

The reviewed isolates (1910–1970) represent an era in the history of medical care hallmarked by major antibiotic discoveries, widespread use of mechanical respiratory techniques in hospitals and the establishment of intensive care units. In order to complete the *Acinetobacter* genomic database, we call for sequencing the whole genomes of ~100 old isolates that have not been sequenced so far (Table S2). Analysing the genomes of these earliest isolates contributes to a better understanding of the current diversity within the genus *Acinetobacter*. It helps recognize major temporal signals in the phylogeny of *Acinetobacter* and might provide novel insights into the potential of particular species to emerge as a notorious pathogen.

## Supplementary material

10.1099/ijsem.0.006983 Uncited Fig. S1.

10.1099/ijsem.0.006983 Uncited Supplementary Material 1.

## Abbreviations

ATCC: American Type Culture Collection
MALDI-TOF: matrix-assisted laser desorption/ionization-time of flight
NCDO: National Collection of Dairy Organisms
NCIMB: National Collection of Industrial, Food and Marine Bacteria
