# Supplementary figures and images for: History of the taxonomy of Acinetobacter: the emergence of hospital-adapted species of global health concern
Source: Int J Syst Evol Microbiol. 2025 Dec 5;75(12):006983. doi: 10.1099/ijsem.0.006983 (PMC12680336; doi:10.1099/ijsem.0.006983)

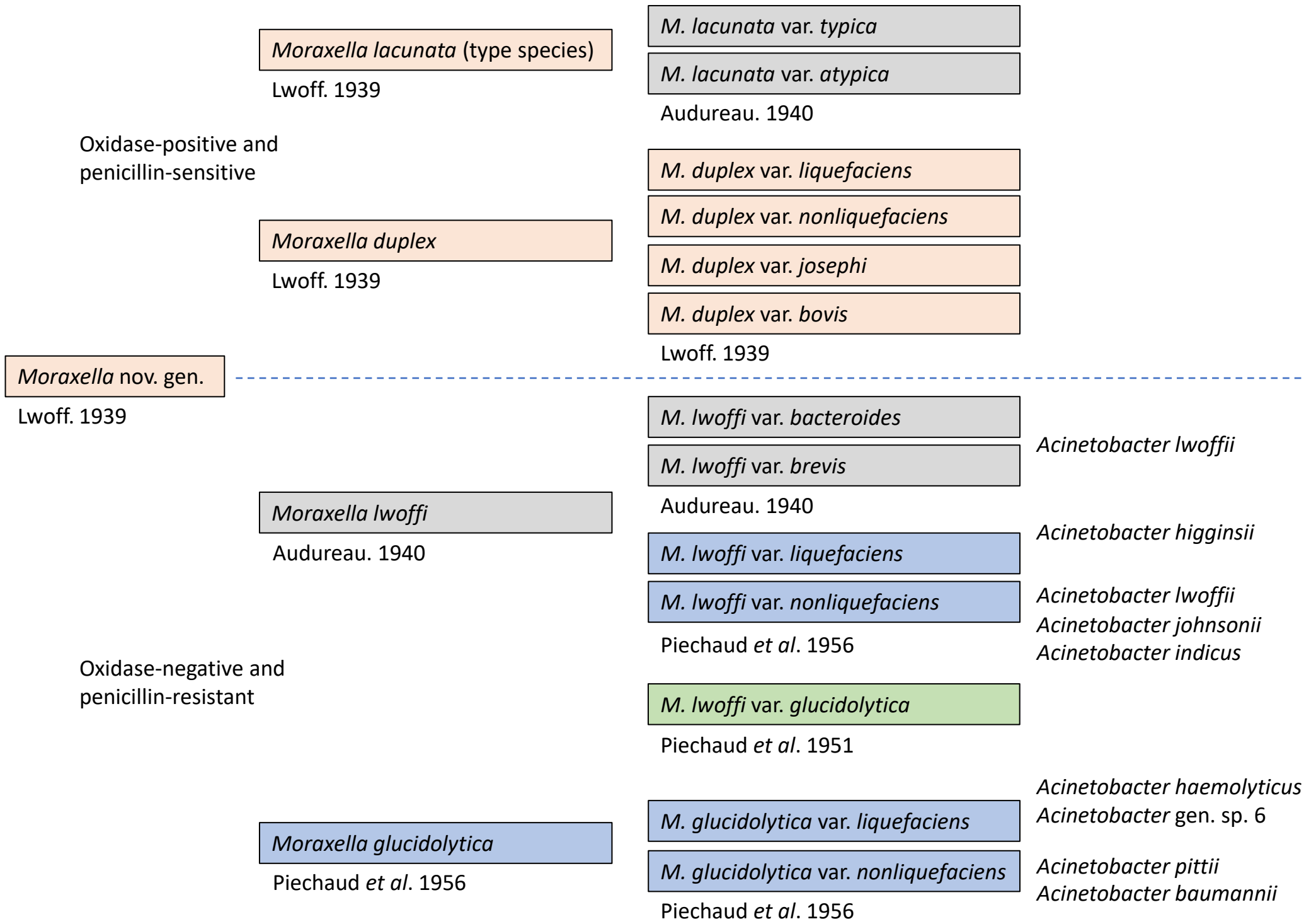

Supplement: Uncited Fig. S1. [file ijsem-75-06983-s001.pdf]
